# SensiScreen^®^ *KRAS* exon 2-sensitive simplex and multiplex real-time PCR-based assays for detection of *KRAS* exon 2 mutations

**DOI:** 10.1371/journal.pone.0178027

**Published:** 2017-06-21

**Authors:** Alice Riva, Michael BØrgesen, Mariann Guldmann-Christensen, Majbritt Hauge Kyneb, Kirsten Voogd, Christina Andersen, Samantha Epistolio, Elisabetta Merlo, Tine Yding Wolff, Stephen Hamilton-Dutoit, Jan Lorenzen, Ulf Bech Christensen, Milo Frattini

**Affiliations:** 1 Laboratory of Molecular Pathology, Institute of Pathology, Locarno, Switzerland; 2 PentaBase Aps, Odense, Denmark; 3 Laboratory of Research and Development, Department of Pathology, Aarhus University Hospital, Aarhus, Denmark; 4 Life Science Division, Danish Technological Institute, Aarhus, Denmark; Rutgers, the State Univesity of New Jersey, UNITED STATES

## Abstract

Activating mutations in codon 12 and codon 13 of the *KRAS* (*Kirsten rat sarcoma viral oncogene homolog*) gene are implicated in the development of several human cancer types and influence their clinical evaluation, treatment and prognosis. Numerous different methods for *KRAS* genotyping are currently available displaying a wide range of sensitivities, time to answer and requirements for laboratory equipment and user skills. Here we present SensiScreen^®^ *KRAS* exon 2 simplex and multiplex CE IVD assays, that use a novel real-time PCR-based method for *KRAS* mutation detection based on PentaBase’s proprietary DNA analogue technology and designed to work on standard real-time PCR instruments. By means of the included BaseBlocker™ technology, we show that SensiScreen^®^ specifically amplifies the mutated alleles of interest with no or highly subdued amplification of the wild type allele. Furthermore, serial dilutions of mutant DNA in a wild type background demonstrate that all SensiScreen^®^ assays display a limit of detection that falls within the range of 0.25–1%. Finally, in three different colorectal cancer patient populations, SensiScreen^®^ assays confirmed the *KRAS* genotype previously determined by commonly used methods for *KRAS* mutation testing, and notably, in two of the populations, SensiScreen^®^ identified additional mutant positive cases not detected by common methods.

## Introduction

*KRAS* belongs to the RAS family of small GTPases involved in the coupling of signal transduction from surface receptors to many different targets that regulate diverse biological responses including cell growth, proliferation, differentiation and survival [1,2]. In cancer, the *KRAS* gene is typically altered by activating point mutations occurring in hot-spots, mainly in codons 12 and 13 of exon 2 (overall accounting for more than 80% of *KRAS* mutations) and, less frequently, in codons 59, 61, 117 and 146 [3–7]. The *KRAS* gene is mutated in several cancers, including adenocarcinomas occurring in the pancreas, lung, ovary and thyroid. However, it is in colorectal cancer (CRC) that *KRAS* mutations have recently acquired most clinical significance [4,8,9]. CRC is the third most diagnosed cancer in both men and women and it is one of the leading causes of cancer related mortality, accounting for more than 600.000 deaths worldwide annually [10–12]. Standard care for patients with metastatic CRC (mCRC) is surgery combined with adjuvant chemotherapy or radiotherapy. However, more recently, targeted therapies have been included in the treatment of selected patients with mCRC. In particular, several studies have clearly and extensively demonstrated that monoclonal antibodies (e.g. cetuximab and panitumumab) targeting the Epidermal Growth Factor Receptor (*EGFR*) may be effective in the treatment of mCRC patients with tumors showing a *KRAS* exon 2 wild type status [8,9,13]. In fact, activating *KRAS* mutations (occurring in about 30–40% of CRC) are associated with lack of response to *EGFR* (*epidermal growth factor receptor*) inhibitors and, at least for panitumumab, have been associated with a detrimental effect with respect to other chemotherapeutic regimens [14]. Based on this knowledge, the recommendations issued by the US Food and Drug Administration (FDA) and the European Medicines Agency (EMA) now mandate mutational testing of *KRAS* in mCRC patient tumors before the administration of *EGFR* inhibitors. Of note, especially in the case of rectal cancer, the tumor material available to laboratories for testing may be limited to only small biopsies, or tissues from partially treated patients containing only few cancer cells, thus making *KRAS* mutational assessment even more challenging. Therefore, sensitive mutation testing techniques have become important tools when selecting patients who will benefit from targeted anti-*EGFR* agents.

The response rates of CRC patients with apparently wild type *KRAS* tumors treated with anti-*EGFR* therapies range from only 40% to 60% [15]. The high numbers of non-responders could, at least in part, be due to intratumoral heterogeneity of *KRAS* mutations that are not included in the tumor areas analysed [16]. However, it may also reflect the fact that there are currently no specific requirements regarding the limit of detection (LOD) for the methods used for *KRAS* mutation testing, in spite of recent evidence indicating that mCRC patient tumors with as few as 1% *KRAS* mutated cancer cells fail to respond to anti-*EGFR* therapies [17]. Thus, direct sequencing (DS) still remains one of the most widely used methodologies for *KRAS* testing, in spite of its low LOD (10–50%) [18,19]. This is the case, even though several more sensitive commercial methods for *KRAS* mutation testing are now available. These include next-generation sequencing (NGS) methods with LODs of 1–10%, depending on platform and coverage [18,20–22], mass spectrometry-based methods with LODs down to 5% [23], and real-time PCR-based methods such as cobas^®^ *KRAS* mutation test (Roche, LOD of 2.4–12.1%) [24], therascreen^®^ *KRAS* RGQ PCR kit (Qiagen, LOD of 0.5–1%) [20], and Idylla™ *KRAS* Mutation Test (Biocartis, LOD ≥5–10%) [25]. Digital droplet PCR (ddPCR) is the latest addition to the technologies available for *KRAS* mutation testing recently demonstrating a LOD as low as 0.0005%, although a large amount of DNA was required to achieve these levels [26]. Thus, the most common methods used for *KRAS* mutation testing have very different LODs with possible implications for cancer treatment in tested patients. Furthermore, these methods are characterized by marked variations in handling times, time to answer, as well as differences in the skills and expertise required of the personnel performing the analyses, the latter being especially relevant with regard to NGS and ddPCR methods.

In this study, we report the development of new real-time PCR-based assays for *KRAS* mutation detection, called SensiScreen^®^ *KRAS* EXON 2 simplex and multiplex, CE IVD. SensiScreen^®^ assays are based on PentaBase’s proprietary DNA analogue technology [27,28], and are designed to work on standard real-time PCR instruments. SensiScreen^®^ was developed with the aim of combining high sensitivity with ease-of-use, short handling time, and short time to answer. Furthermore, the SensiScreen^®^ multiplex version allows for fast screening of the 9 most common *KRAS* mutations (7 single- and 2 double-mutations) with minimal use of input DNA. We compared, SensiScreen^®^ assays in different cohorts of CRC patients with DS, mutant-enriched PCR (ME-PCR), therascreen^®^ and cobas^®^ genotyping methods. In addition to confirming all mutations found by the alternative methods, SensiScreen^®^ assays identified additional *KRAS* exon 2 mutated cases in two out of three patient populations studied.

## Materials and methods

### Construction of model templates

For the development of SensiScreen^®^, seven plasmids comprising sequences with 7 different *KRAS* exon 2 mutations (G12A: *c*.*35G>C*, G12D: *c*.*35G>A*, G12R: *c*.*34G>C*, G12C: *c*.*34G>T*, G12S: *c*.*34G>A*, G12V: *c*.*35G>T* and G13D: *c*.*38G>A*) were constructed. A 221 bp fragment of the *KRAS* gene surrounding the codon 12/13 hotspot was cloned from a wild type (WT) genomic DNA template using the primers *Cloning forward* and *Cloning reverse* [29] (S1 Table). The fragment was inserted into the *pCR4TOPO-vector* according to the manufacturer’s instructions (Invitrogen, Carlsbad, CA, USA). This WT plasmid was used as template in a series of site-directed mutagenesis reactions using designed mutagenesis primers (PentaBase ApS, Odense, Denmark) and the QuikChange Lightning Site-Directed Mutagenesis Kit (Agilent Technologies, Santa Clara, CA, USA) for introduction of the 7 most common *KRAS* exon 2 mutations. The resulting plasmids were validated by sequencing using the vector specific primers *M13rev* and *M13fwd*. The plasmids were purified using GenElute Plasmid Miniprep Kit (Sigma-Aldrich, St. Louis, MO, USA) and linearized with the restriction enzyme *Not*I (New England Biolabs, Ipswich, MA, USA). The quality of the linearized plasmids was evaluated by agarose gel electrophoresis and quantitated using a Nanodrop (Thermo Scientific Waltham, MA, USA) spectrophotometer.

### Cell lines

Genomic DNA from six tumor cell lines, SW480, SW1116, MICOL29, T84, CAL-62 and A549, containing 6 different *KRAS* mutations were used for sensitivity studies (S2 Table). The cell lines were subcultured in appropriate media according to the manufacturer’s instructions and genomic DNA was isolated using the QIAmp Mini kit (Qiagen, Chatsworth, CA, USA). SW480 (catalogue number: CCL-228™), SW1116 (catalogue number: CCL-233™), T84 (catalogue number: CCL-248™) and A549 (catalogue number: CCL-185™) were obtained from ATCC^®^; CAL-62 (catalogue number: ACC-448™) was obtained from DSMZ^®^ and MICOL29 was kindly provided by Fondazione IRCCS Istituto Nazionale dei Tumori, Milan, Italy.

### SensiScreen^®^ assay development

SensiScreen^®^ real-time PCR assays were developed to specifically amplify few copies of mutated DNA with a single-nucleotide polymorphism in a large WT background. In order to achieve this, we took advantage of PentaBase’s intercalating nucleic acid modifications called pentabases™ that allow for the creation of oligonucleotides that, among other features, show increased affinity and specificity towards their target sequence [27,28]. Thus, SensiScreen^®^ include primers, hydrolysis probes and blocking oligonucleotides containing pentabases™, which are designated SuPrimers™, HydrolEasy™ probes and BaseBlockers™, respectively. Different oligo designs containing different combinations of pentabases™ were tested and evaluated for: specificity of BaseBlockers™, signal-to-noise ratio of HydrolEasy™ probes, and PCR efficiency of SuPrimers™. Furthermore, to obtain the highest possible specificity towards the selected mutations, we evaluated different mutation-specific primer designs all with the particular sequence variation in the 3’ end of the primer, and with either no additional mismatch or one of the three possible nucleotide variations in position -3 or -4 relative to the 3’-end. All SensiScreen^®^ assays (7 simplex and 2 multiplex) also include an internal control assay targeting the *CYP17A1* gene that does not interfere with amplification by the primary assays (data not shown).

### Sensitivity studies

In order to evaluate the LOD of SensiScreen^®^, we performed serial dilutions of mutated DNA from cell lines and plasmid DNA (*pCR4TOPO-G12C*), respectively, in a background of WT DNA (Promega, Madison,WI, USA) totalling 50 ng genomic input DNA. Eight different concentrations of mutated DNA (10%, 5%, 1%, 0.5%, 0.1%, 0.05%, 0.01% and 0%) were tested by real-time PCR, using the SensiScreen^®^ protocol described below.

### Patient samples

Inclusion criteria for the study were a diagnosis of CRC following surgical resection and the availability of at least 3 different tissue blocks containing cancer cells for each patient. The patients had undergone routine surgery and clinico-pathological assessment of their tumors as part of their standard clinical care. The tumor materials used in the study were surplus to requirements for routine testing. All materials were anonymized. This study was approved by the Institutional Ethical Committee of the Institute of Pathology of Locarno (Switzerland), by the Central Denmark Region Committee on Health Research Ethics, and by the Danish Data Protection Agency. All procedures were performed in accordance with the ethical standards of the Helsinki Declaration.

### Tissue analyses

Formalin-fixed paraffin-embedded (FFPE) tumor blocks were analysed for quality and tumor content. A single representative tumor block from each patient, containing at least 70% neoplastic cells (if necessary after macrodissection to enrich tumor cell content), was selected. Genomic DNA was extracted from six 7 μm-thick serial sections of each FFPE block using the QIAmp Mini kit (cohort 1) and QIAsymphony (cohort 2+3) (Qiagen) according to the manufacturer’s instructions.

**Cohort 1:** FFPE samples from 100 patients with histologically confirmed CRC collected from 1996 to 2009 were retrospectively analysed for *KRAS* exon 2 mutations by DS, ME-PCR and SensiScreen^®^ simplex and multiplex. All tumors were colorectal adenocarcinomas, diagnosed at the Institute of Pathology in Locarno, Switzerland (S3 Table).

**Cohort 2:** FFPE samples from 79 patients with histologically confirmed CRC were retrospectively analysed for *KRAS* exon 2 mutations by DS, therascreen^®^ (Qiagen) and SensiScreen^®^ simplex. All tumors were colorectal adenocarcinomas, diagnosed at Aarhus University Hospital, Denmark (S4 Table).

**Cohort 3:** FPPE samples from 283 patients with histologically confirmed CRC were retrospectively analysed for *KRAS* exon 2 mutations by cobas^®^ (Roche, Basel, Switzerland) and SensiScreen^®^ multiplex. Cases only found to be mutated with SensiScreen^®^ Multiplex were in addition analysed with SensiScreen^®^ simplex. All tumors were colorectal adenocarcinomas, diagnosed at Aarhus University Hospital, Denmark (S5 Table).

### Mutational analysis by direct sequencing

The mutational analyses of *KRAS* exon 2 by DS were performed at the Institute of Pathology in Locarno (Switzerland) using the primers listed in S1 Table. Samples were subjected to automated sequencing on an ABI PRISM 3130 Genetic Analyzer (Applied Biosystems, Foster City, CA, USA), and evaluated with Sequencing Navigator Software (Applied Biosystems). All mutated cases were confirmed twice with independent PCR reactions.

### Mutational analysis by ME-PCR

Analyses of *KRAS* exon 2 mutations by ME-PCR were performed at the Institute of Pathology in Locarno (Switzerland) essentially as described previously [30,31]. ME-PCR combines PCR amplification with restriction enzyme digestion to enrich for mutant alleles, while WT alleles are eliminated by digestion [32–34]. ME-PCR products were subsequently subjected to automated sequencing on an ABI PRISM 3130 Genetic Analyzer. All mutated cases were confirmed twice with independent PCR reactions.

### Mutational analysis by therascreen^®^

The analysis of *KRAS* mutations in exon 2, codon 12/13 and 61 using the therascreen^®^ *KRAS* test (Qiagen) was performed at the Department of Pathology, Aarhus (Denmark). DNA was extracted from FFPE tissue using the QiaSymphony (Qiagen) according to the manufacturer’s instructions. The DNA concentration was measured (Implen, Munich, Germany) and the DNA was diluted to a concentration of 5 ng/μl. Five μl were added to each reaction tube. The analysis was subsequently run on a Rotor-Gene Q (Qiagen).

### Mutational analyses by cobas^®^ *KRAS* mutation test

A Cobas 480Z (Roche) was used for the testing of *KRAS* mutations in exon 2 codons 12/13 and 61. The analysis was performed at the Department of Pathology, Aarhus. Extraction and measurement of the DNA concentration was performed as described above (therascreen^®^). The DNA was diluted to 5 ng/μl. Twenty-five μl were added to each reaction tube.

### Mutational analysis by SensiScreen^®^

SensiScreen^®^ development and clinical validation was performed in 25 μl reactions in KAPA Probe Fast qPCR 2x Master Mix (KAPA Biosystems, Wilmington, MA, USA) on a Rotor-Gene 6000 (Corbett Research, Mortlake, NSW, Australia), a CFX96 (Bio-rad Laboratories, Hercules, CA, USA) and a Mx3005P qPCR system (Stratagene, CA, USA). The reaction mixture contained 300–900 nM of each primer, 200 nM of each probe, and 1000–5000 nM WT BaseBlocker (WTB). qPCR (quantitative PCR) was performed using either 50 ng of cell line DNA or 50 ng WT DNA (Promega G304A) in the absence or presence of approximately 1000 copies of plasmid DNA containing the indicated mutations. The thermocycling conditions used were: 2 min of initial activation of the hotstart taq-polymerase at 95°C, followed by 45 cycles of a 2-step PCR with a 15 sec denaturation step at 94°C and 60 sec extension step at 60°C. Fluorescence was measured at the end of each extension step.

The qPCR threshold cycle (Ct) of normalized fluorescence was used for the evaluation of the data. Ct is defined as the number of cycles where a fluorescence signal crosses the threshold. In order to make data analysis independent of the type of instrument used, the threshold was defined as 10% of the signal strength of the reference assay at cycle 45 (S1 Fig). For all valid samples (23 < Ct_ref_ < 36), a ΔCt value was calculated by taking the Ct value of the mutation-specific assay and subtracting the Ct value of the reference assay:

$$\Delta Ct={Ct}_{mutation}-{Ct}_{reference}$$

Patient samples analysed by SensiScreen^®^ were regarded as positive for a given mutation if the Ct_mutation_ was ≤ 38 and ΔCt was ≤9.

## Results

### SensiScreen^®^ assay design and specificity

**Fig 1:**
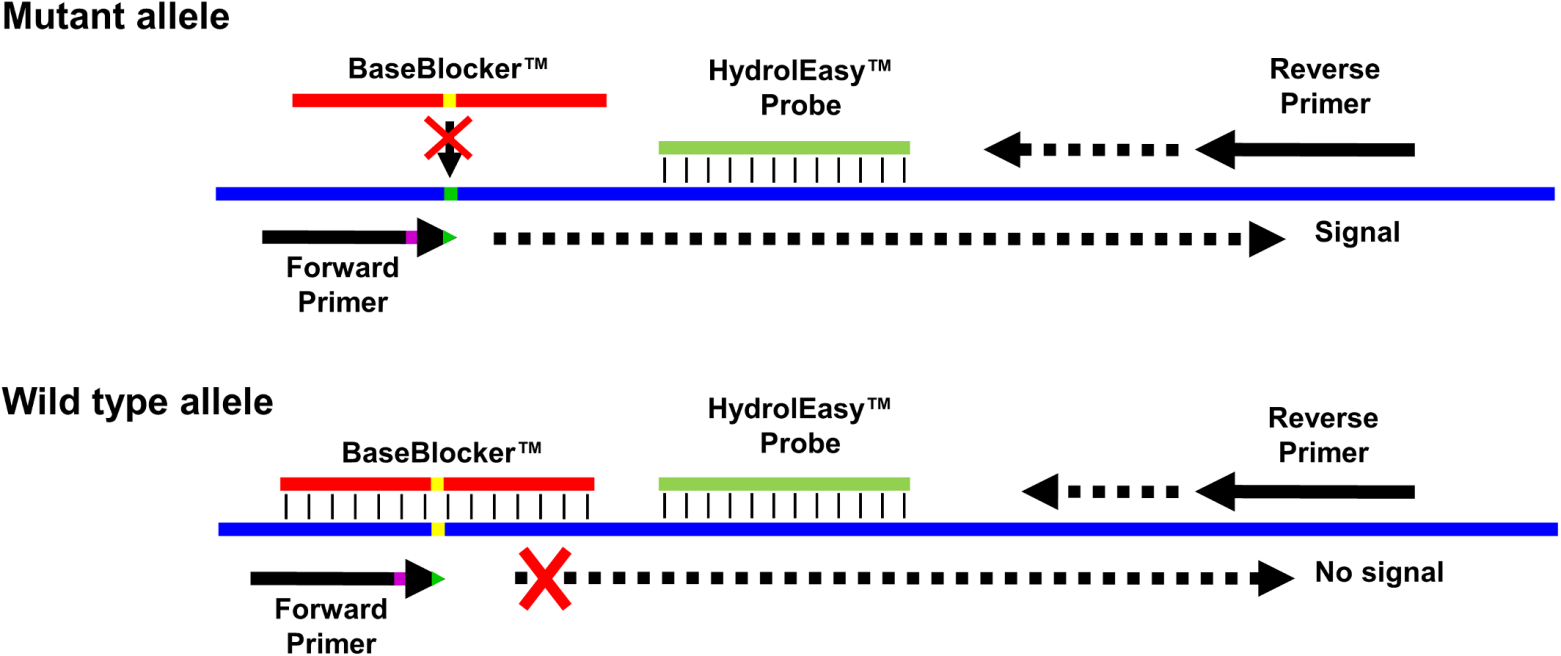
SensiScreen^®^ assay design. SensiScreen^®^ *KRAS* exon 2 features 7 mutation-specific assays as well as a common reference assay. All assays include a common reverse primer and a green fluorescent HydrolEasy™ probe. The reference assay features an allele-independent forward primer (not shown), while the mutation-specific assays include allele-specific forward primers and a BaseBlocker™. The allele-specific forward primers include the particular sequence variation in the 3´-end (green) and can include an additional sequence variation in position +3 or +4 relative to the 3´-end (purple). The BaseBlocker™ is complementary to the wild type sequence (yellow) and specifically blocks amplification of the wild-type allele.

**Fig 2:**
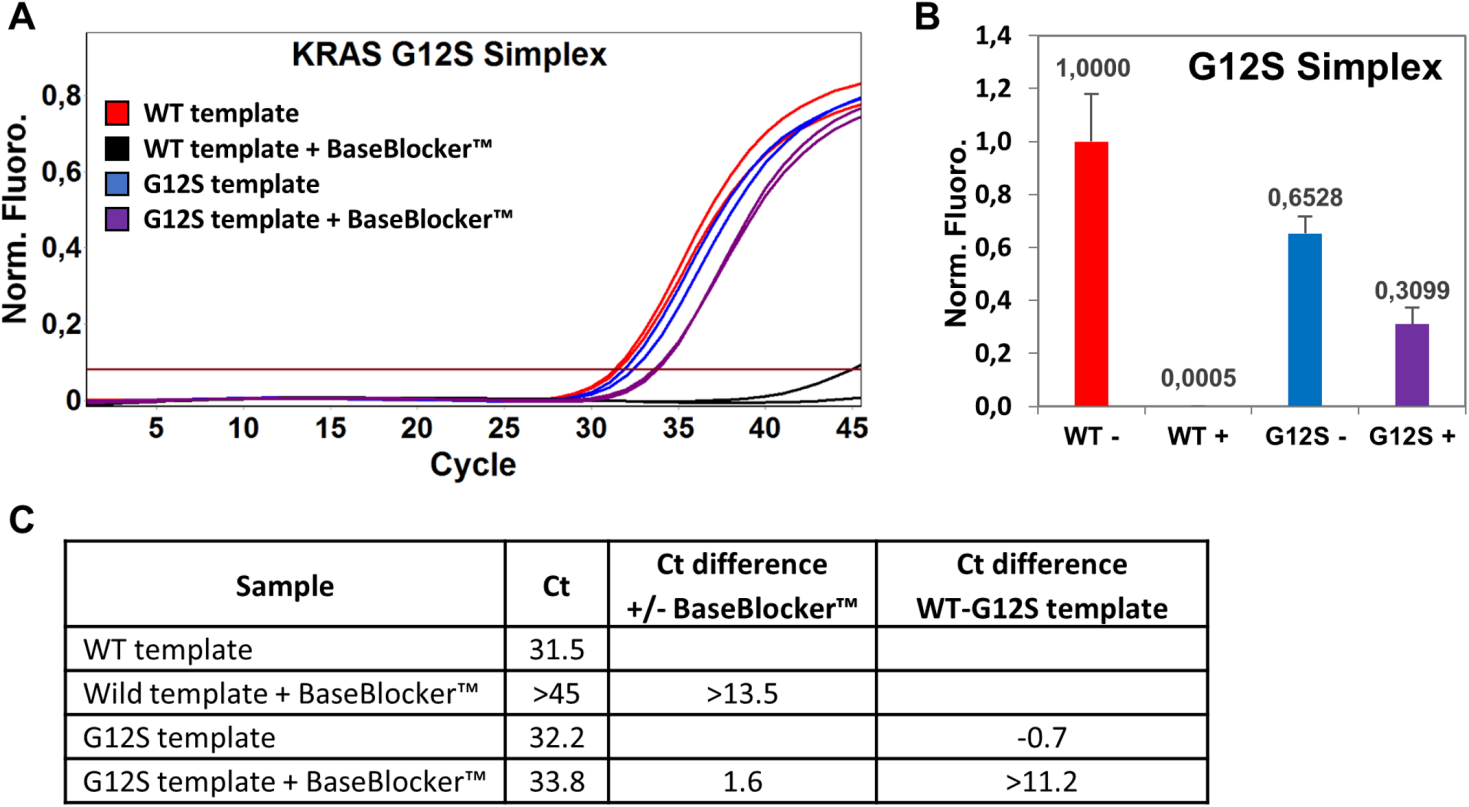
Addition of a BaseBlocker™ strongly increases SensiScreen^®^ assay specificity. (A) Normalized real-time PCR amplification plot showing the specificity of SensiScreen^®^ G12S simplex assay in the absence and presence of a wild type blocking BaseBlocker™. (B+C) The inclusion of a BaseBlocker™ (+) impairs amplification of the wild-type allele by 99.95% (B) and increases the difference in threshold cycle (Ct) value between wild-type (WT) and mutant (G12S) DNA samples from -0.7 to >11.2 (C). SensiScreen^®^ *KRAS* G12S Simplex real-time PCR ±2000 nM of BaseBlocker™ was performed on a Corbett Rotor-Gene 6000 instrument using 50 ng of wild type (WT) human genomic DNA and approximately 500 copies of *KRAS* G12S template. PCR amplification plot (A) is representative of three independent experiments. Bars (B) represent the mean +S.D. of three independent experiments.

SensiScreen^®^ *KRAS* EXON2 simplex and multiplex CE IVD (hereinafter referred to as SensiScreen^®^ simplex and multiplex) are real-time PCR-based assays developed by PentaBase (Denmark) for the detection of *KRAS* exon 2 mutations. SensiScreen^®^ detects nine different mutations (seven single and two double nucleotide polymorphisms) in exon 2 codons 12 and 13 of the *KRAS* gene (Table 1) and is certified in accordance to the EU guidelines 98/79/EC Medical equipment for *in vitro* diagnostics. The simplex version includes a reference assay and 7 different mutation-specific assays, while the multiplex version includes a reference assay and 2 different mutation-specific assays (Table 1). The reference assay comprises a set of allele-independent primers and a green fluorescent HydrolEasy™ probe, while the mutation-specific assays include an allele-independent forward primer, allele-specific reverse primers, a green fluorescent HydrolEasy™ probe and a BaseBlocker™ (Fig 1 and S1 Table). In addition, all assays include an internal control assay comprising a set of allele-independent primers and a yellow fluorescent HydrolEasy™ probe targeting the *CYP17A1* gene. SensiScreen^®^ includes standard DNA oligonucleotides as well as DNA oligonucleotides modified with pentabases™ which are flat hetero aromatic molecules inserted into the backbone of the oligonucleotide via a linker [27,28]. The presence of pentabases™ increases both the affinity, sensitivity and specificity of the oligonucleotide towards the target sequence [27,28]. A central feature of SensiScreen^®^ is the inclusion of a BaseBlocker™ that comprises several pentabases™ and is designed to bind strongly and specifically to WT DNA with little or no affinity towards mutated DNA (Fig 1). Thus, by combining the use of a BaseBlocker™ with allele-specific primers, SensiScreen^®^ qPCR leads to highly specific amplification of mutated DNA whereas amplification of WT DNA is strongly impaired (Fig 2).

**Table 1:** *KRAS* mutations detected by SensiScreen^®^ *KRAS* exon 2 simplex and multiplex.

*KRAS* CDS mutation	*KRAS* AA substitution	COSMIC ID	SensiScreen multiplex assay
*c*.*35G>C*	Gly12Ala (G12A)	COSM522	Multiplex 2
*c*.*35G>A*	Gly12Asp (G12D)	COSM521	Multiplex 2
*c*.*34G>C*	Gly12Arg (G12R)	COSM518	Multiplex 1
*c*.*34G>T*	Gly12Cys (G12C)	COSM516	Multiplex 1
*c*.*34G>A*	Gly12Ser (G12S)	COSM517	Multiplex 1
*c*.*35G>T*	Gly12Val (G12V)	COSM520	Multiplex 1
*c*.*38G>A*	Gly13Asp (G13D)	COSM532	Multiplex 2
*c*.*34_35GG>TT*	Gly12Phe (G12F)	COSM512	Multiplex 1
*c*.*34_35GG>AT*	Gly12Ile (G12I)	COSM34144	Multiplex 1

*KRAS*, Kirsten rat sarcoma viral oncogene homolog; CDS, COSMIC database sequence; AA, amino acids; COSMIC, catalogue of somatic mutations in cancer; COSMIC ID, COSMIC identification number.

### SensiScreen^®^ assay sensitivity

**Fig 3:**
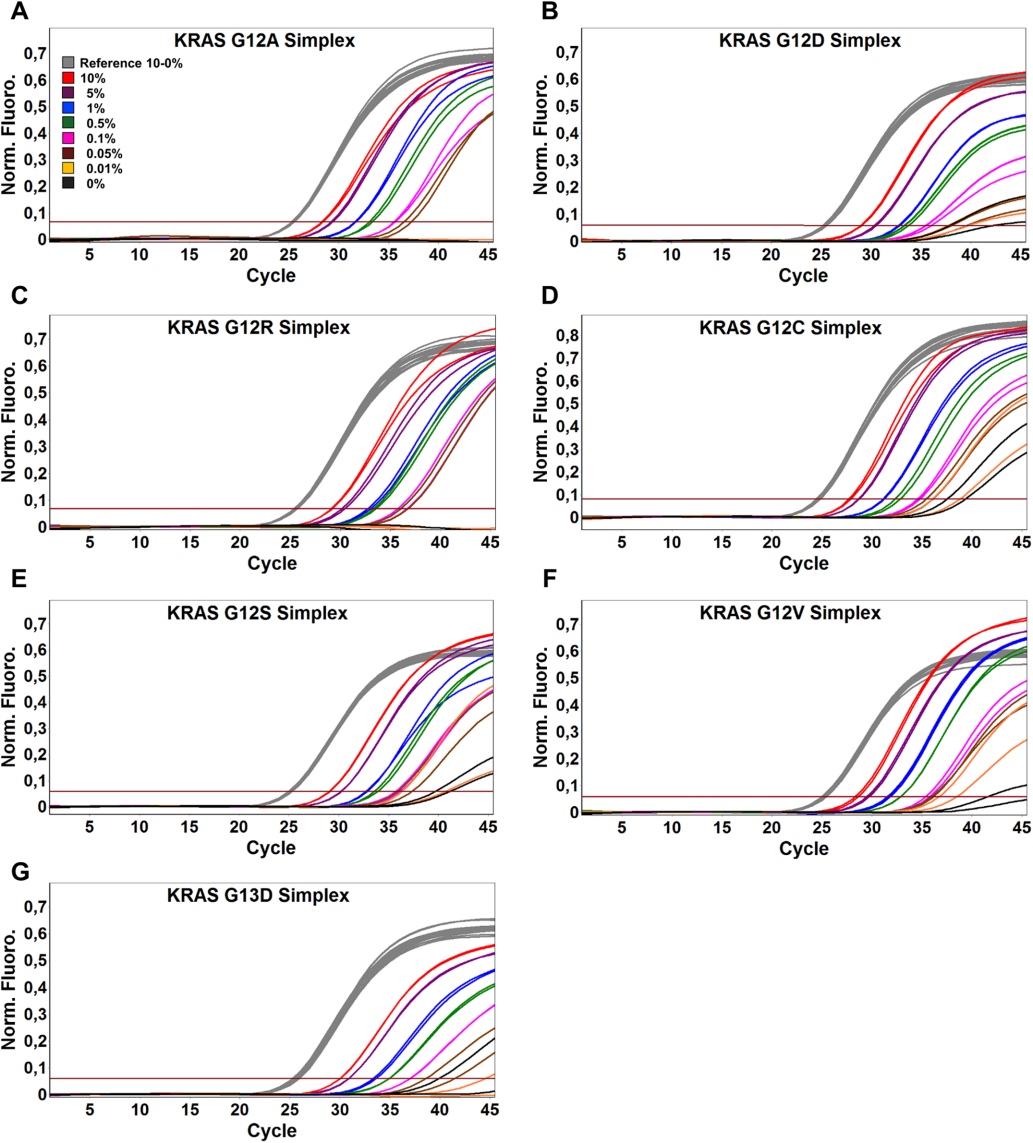
Rotor-Gene 6000 PCR amplification plots of SensiScreen^®^ *KRAS* exon 2 simplex assays using serial dilutions of mutated DNA in a wild type background. 50 ng and/or approximately 16,000 copies of DNA was added to each reaction. The threshold was set at 10% of the average fluorescence signal of the reference assay at cycle 45. Legend describes the fraction of cell line DNA and/or mutated copies of KRAS exon 2 templates.

**Fig 4:**
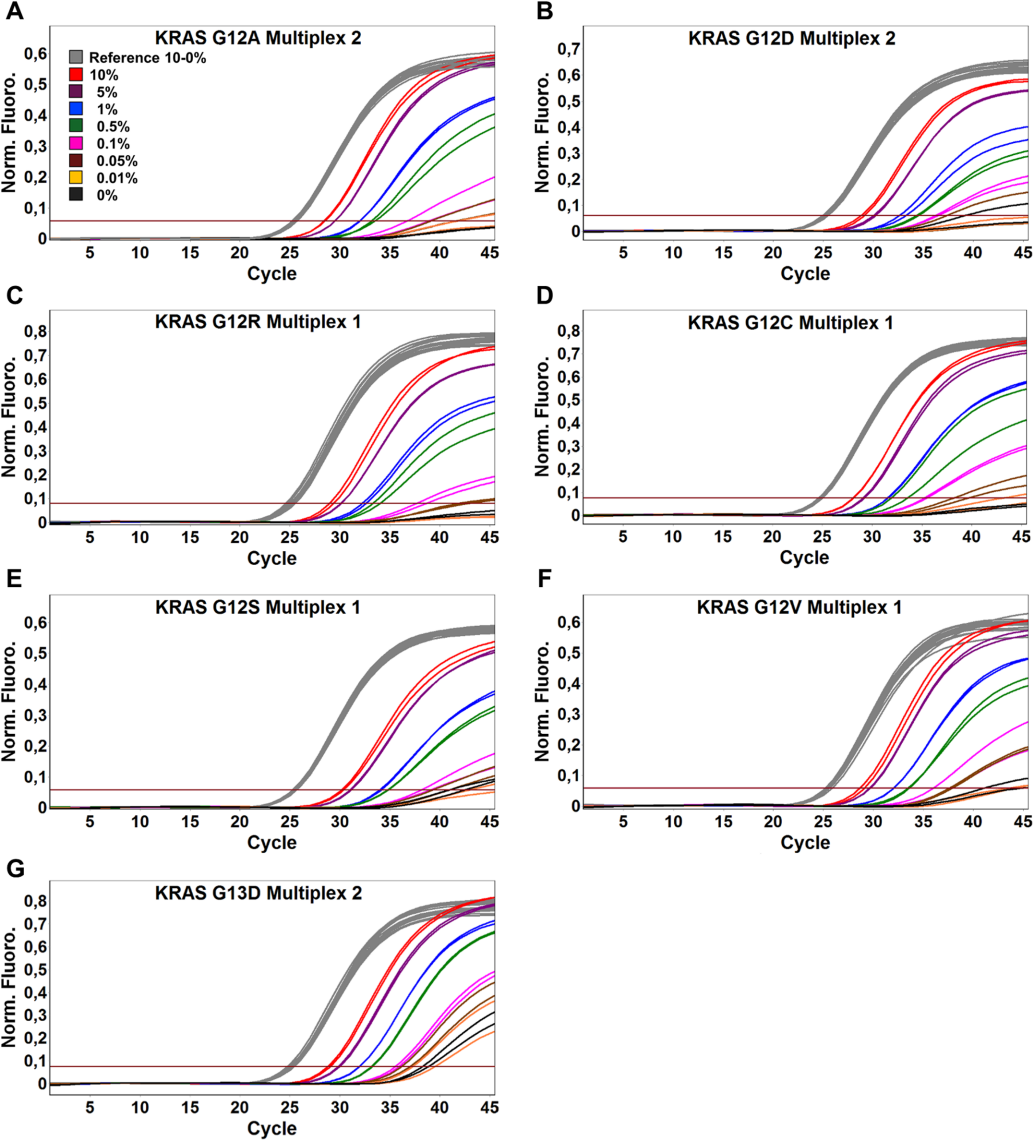
Rotor-Gene 6000 PCR amplification plots of SensiScreen^®^ *KRAS* exon 2 multiplex assays using serial dilutions of mutated DNA in a wild type background. 50 ng and/or approximately 16,000 copies of DNA was added to each reaction. The threshold was set at 10% of the average fluorescence signal of the reference assay at cycle 45. Legend describes the fraction of cell line DNA and/or mutated copies of KRAS exon 2 templates.

We sought to develop an assay that was not only highly specific for amplification of mutant DNA but also sensitive enough to be used on samples that contain very low amounts of mutated DNA. Thus, to evaluate the sensitivity of SensiScreen^®^ simplex and multiplex assays, we performed serial dilutions of mutant cell line or plasmid DNA in a WT background. To evaluate the robustness of SensiScreen^®^, the sensitivity studies were performed on two different real-time PCR systems, the Rotor-Gene 6000 (Corbett Research) (Figs 3 and 4, Table 2) and the MyGo Pro (IT-IS Life Science Ltd., Mahon, Ireland) (S2 Fig; S3 Fig and Table 2).

**Table 2:** Sensitivity and PCR efficiency of SensiScreen^®^ simplex and multiplex assays determined by serial dilutions of mutated DNA in a wild type background. 50 ng and/or approximately 16,000 copies of DNA was added to each real-time PCR mixture. The threshold was set at 10% of the average fluorescence signal of the reference assay at cycle 45.

	Rotor-Gene			MyGo Pro		
Assay	LOD*	ΔCt WT^	Efficiency (%)^¤^	LOD*	ΔCt WT^	Efficiency (%)^¤^
G12A Spx	0.5%	No Signal	88	0.5%	No Signal	96
G12D Spx	0.5%	12.4	94	0.5%	16.6	99
G12R Spx	0.5%	No Signal	97	0.5%	No Signal	97
G12C Spx	0.5%	13.6	86	0.5%	13.8	98
G12S Spx	0.5%	14.5	89	0.5%	No Signal	96
G12V Spx	0.5%	19.3	101	0.5%	No Signal	95
G13D Spx	0.5%	14.2	85	0.25%	14.7	106
G12A Mpx 2	0.5%	No Signal	86	0.5%	12.6	102
G12D Mpx 2	1.0%	13.85	74	1.0%	No Signal	96
G12R Mpx 1	0.5%	No Signal	97	1.0%	No Signal	80
G12C Mpx 1	0.5%	No Signal	92	1.0%	No Signal	91
G12S Mpx 1	1.0%	15.6	87	1.0%	17.4	96
G12V Mpx 1	0.5%	16	92	0.5%	No Signal	93
G13D Mpx 2	0.25%	12.4	100	0.25%	No Signal	109

LOD, limit of detection; WT, wild type; SPX, simplex assay; MPX, multiplex assay.

* Limit of detection (LOD) determined using a ΔCt between reference and assay of 9.

^ ΔCt calculated as the lowest possible difference in Ct between reference and assay of the duplicate PCR reactions performed using the 0% dilution point.

¤ PCR efficiency calculated using the 10%, 5%, 1% and 0.5% dilution points.

SensiScreen^®^ determines if a sample is positive or negative for the specific mutation(s) by measuring the difference in cycle threshold (ΔCt) of the reference assay (Ct_reference_) and the cycle threshold of the mutation-specific assay (Ct_mutation_). The ΔCt cut-off should be set at a value that will avoid false positive results from amplification of WT samples and at the same time identify as many true positive samples as possible. Since a ΔCt of 12.4 was the lowest possible value identified when using WT DNA as template, we considered a ΔCt of 9 as the optimal value for subsequent analysis of assay sensitivities.

Interestingly, using a ΔCt of 9, the LODs of the different SensiScreen^®^ assays were very similar on the two real-time PCR instruments. Thus, all SensiScreen^®^ simplex assays except G12R Simplex (LOD less than 1.0% on the MyGo Pro instrument) were found to display a LOD of less than 0.5%. Similarly, using the same ΔCt of 9, SensiScreen^®^ multiplex assays showed LODs from 0.25% (G13D) to 1% on both instruments.

### SensiScreen^®^ assays identify more patients with *KRAS* mutations than commonly used methods

**Fig 5:**
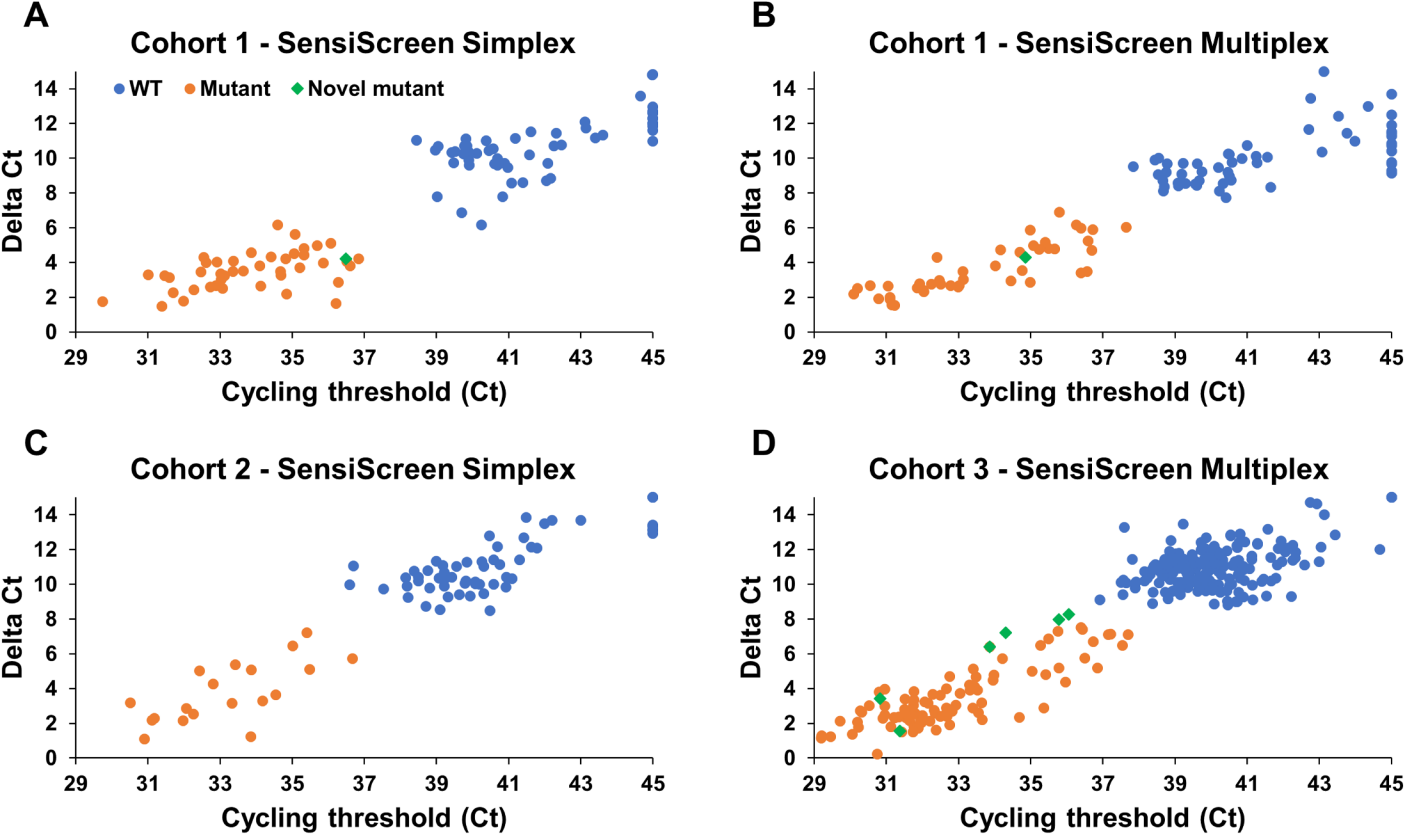
Dot plots of SensiScreen^®^ clinical data. (A+C) Patient samples were analysed for KRAS exon 2 mutations using SensiScreen^®^ simplex. (B+D) Patients were also analysed for KRAS exon 2 mutations using SensiScreen^®^ multiplex. Samples were regarded as mutant if Delta Ct (DCt) values were ≤9 and Ct values were ≤38. Samples with no Ct values for the mutation assay were plotted as Ct = 45. Novel mutant indicates samples that were not identified as mutant by other methods.

To validate SensiScreen^®^ assays in the clinical setting, we used SensiScreen^®^ to retrospectively analyse FFPE DNA from three different patient populations with histologically confirmed CRC that were previously analysed by common KRAS mutation tests. In general, when including both Ct and Delta Ct values in the analysis of the patient samples, there was a good separation of the wild type and mutant groups in all three cohorts (Fig 5). Using the analysis settings described in Table 3, in all three populations, SensiScreen^®^ confirmed the same mutations already identified by the common method(s) used in each population (Table 3; S6 Table, S7 Table and S8 Table). Thus, in cohort 1, both SensiScreen^®^ *KRAS* EXON 2 simplex and multiplex assays confirmed the 28 and 43 mutational cases identified by DS and ME-PCR, respectively (Fig 5, Table 3 and S6 Table). In cohort 2, SensiScreen^®^ *KRAS* EXON 2 simplex confirmed the 17 and 19 mutational cases identified by DS and therascreen^®^, respectively (Table 3 and S7 Table). Finally, in cohort 3, SensiScreen^®^ *KRAS* EXON 2 multiplex confirmed the 87 mutational cases identified by cobas^®^ (Table 3 and S8 Table). Interestingly, however, SensiScreen^®^ assays in addition identified one new mutational case in cohort 1, and 6 new mutational cases in cohort 3. Additional mutational cases identified by SensiScreen^®^ were confirmed SensiScreen^®^ simplex or multiplex when possible.

**Table 3:** Summary of cohort data. Samples analysed with SensiScreen^®^ were regarded as mutant if Delta Ct (ΔCt) values were ≤9 and Ct values were ≤38.

Comparison of SensiScreen^®^ to common methods for *KRAS* exon 2 mutation testing							
	Common method	Cohort no	Total patient samples	Mutated cases identified by		Additional mutated cases identified by SensiScreen^®^	
				Common method	SensiScreen^®^	no	%
***KRAS* exon 2 simplex**	Direct sequencing	1	100	28	44	16	57%
	ME-PCR	1	100	43	44	1	2%
	Direct sequencing	2	79	17	19	2	12%
	therascreen^®^	2	79	19	19	0	0%
***KRAS* exon 2 multiplex**	cobas^®^	3	283	87	93	6	7%

*KRAS*, Kirsten rat sarcoma viral oncogene homolog; no: number; ME-PCR: mutant-enriched PCR.

## Discussion

The assessment of *KRAS* mutational status has acquired high relevance in recent years and, at the same time, has become a challenge. Although originally considered as an alteration occurring in early phases of developing CRC, *KRAS* mutations may also appear first in the metastatic lesion, probably due to the selection of a clone already present in the primary tumor. The fraction of tumoral DNA in the sample is highly dependent on which tissue block the pathologist selects for further analysis [35], and tumor heterogeneity may significantly complicate this pathological evaluation. Furthermore, tumoral cells can be polysomic, and the allele with the *KRAS* WT sequence can be overrepresented with respect to the allele carrying the *KRAS* mutation. Finally, the availability of either only small biopsy samples with few cancer cells or tumors already treated prior to surgical resection (as is often the case with rectal carcinomas), presents an additional challenge for diagnostic laboratories. It is clear that the availability of methodologies that are both highly sensitive and specific may make a significant contribution to the successful evaluation of the *KRAS* mutational status in the routine clinical setting. An incorrect *KRAS* mutational analysis may lead either to the use of inappropriate targeted therapy or to a delay in the most suitable treatment, both of which may have serious consequences for the patients involved. Furthermore, it has been proposed that patients with tumors characterized by a low level of *KRAS* mutated DNA may also be resistant to the administration of *EGFR*-targeted therapies, although the precise threshold of mutant allele required for *EGFR*-targeted therapy resistance has not yet been established [17,36]. Fortunately, recently developed methods for *KRAS* mutation testing, including SensiScreen^®^, can detect highly diluted mutant DNA. Thus, we show here, that SensiScreen^®^, a novel real-time PCR based assay for detection of *KRAS* exon 2 mutations, is extremely sensitive and is able to find more mutation positive patients than the commonly used methods we compared it with.

One of the enhanced qualities of SensiScreen^®^ is its specificity. This is mainly related to the inclusion of a blocking oligonucleotide (BaseBlocker™) that is designed to bind strongly and specifically to WT DNA in order to inhibit its amplification. Thus, compared with standard ARMS PCR, the BaseBlocker™ provides a large part of the allele specificity of the assay that is normally solely dependent on the mutation-specific primers. This not only leads to a better overall specificity of the SensiScreen^®^ assays, but also allows for the use of primers where the efficiency is not compromised by primer specificity design strategies such as additional mismatches. In line with this, most SensiScreen^®^ assays display a sensitivity that is close to the theoretical limit of a PCR with 100% efficiency and, importantly, is associated with no or very low amplification of the WT template. Thus, by performing serial dilutions of mutant DNA, we show that SensiScreen^®^ simplex and multiplex assays can safely detect as little as 0.25–1% mutant DNA in a WT background. Finally, the fact that the assays perform very similar on both the air temperature-controlled slow ramping Rotor-Gene machine and the pure silver block fast ramping MyGo Pro machine underlines the robustness of the SensiScreen^®^ assays.

The sensitivity studies furthermore illustrate the LOD potential of the SensiScreen^®^ technology. Because of the subdued amplification of the WT template, at least for some of the SensiScreen^®^ assays, the ΔCt could potentially be expanded to achieve a LOD down to 0.01% (G12V simplex). Since 50 ng of human genomic DNA corresponds to roughly 16,000 copies, the 0.01% dilution point in theory is equal to just 1.6 copies (i.e. 1–2 copies) of mutant DNA. While the clinical relevance of such a low amount of mutated DNA in primary tumor biopsies is unknown, this degree of sensitivity could be highly relevant when analysing liquid biopsies.

In line with the demonstrated high sensitivity of SensiScreen^®^, when comparing with common methodologies using three separate CRC cohorts, we found that SensiScreen^®^ not only confirmed the mutated samples identified by the common methods, but also identified additional novel mutant cases. This was especially the case when comparing to DS even though the percentages of mutations detected by DS were in accordance with the literature [37,38]. However, also when comparing to the very sensitive ME-PCR method SensiScreen^®^ was able to identify an additional mutant case. Finally, in the largest cohort SensiScreen^®^ multiplex was able to identify additional mutant cases compared to cobas^®^. With regard to the cases classified as mutated only by SensiScreen^®^, since we could not confirm these mutations with other methods, both the simplex and the multiplex version were used to confirm the results when possible.

On a general note, we found in the clinical studies that the WT signals had lower Ct and ΔCt values and thus were closer to the cut off than what we found in the sensitivity studies using cell lines and plasmids. We believe that these putative WT signals are related by an unknown mechanism to the FFPE preparation. This assumption is supported by preliminary studies using SensiScreen^®^ on non-FFPE liquid biopsies in which WT signals generally show higher ΔCt values (data not shown). More tests on liquid biopsies will be needed to confirm this observation, which will allow for the possibility of using a higher ΔCt cut-off of SensiScreen^®^ assays and, thus will lead to a potentially even higher sensitivity for the assays, something that is of particular importance when analysing liquid biopsies. Another point to note is that in several cases, due to low concentration of DNA in the sample (reference >Ct 30), we found that the Ct of the mutation-specific assays and multiplexes was just above 38 while the ΔCt was below 9. Some of these cases could potentially be mutation positive if more material was used, but unfortunately, in most cases it was not possible to repeat the analyses with more DNA due to lack of material.

In addition to its high sensitivity, the advantages of SensiScreen^®^ include a fast turn-around time (less than 2 hours, from PCR preparation to the final result), the same cut-off values valid for all the mutation types (rendering the assay highly user-friendly) and flexibility including the possibility to use whatever real-time PCR machine is available (the results of discrepant cases have been tested using different real-time PCR instruments). The multiplex version represents an innovative solution. Thus, it has as high a sensitivity as the simplex version, and since as little as 5 ng genomic DNA can be used as input, this enables the laboratories to perform a fast screening using only 15 ng of genomic DNA (3 tubes). This allows for a 2-step approach of initial screening with the multiplex assay, followed by confirmation of the result with the simplex assay together with the precise characterization of the mutation. This is a robust and rapid workflow enabling test laboratories to characterize *KRAS* exon 2 status in a few hours.

In conclusion, we have developed a new real-time based assay capable of identifying more patient tumors with mutations in *KRAS* exon 2 (codons 12 and 13) compared with methods commonly used in diagnostic laboratories for detection of mutations in this gene. SensiScreen^®^ has a high specificity and sensitivity, is time saving, and can also be applied in other cancer types, particularly in regards to tumors in which *KRAS* mutational status analysis is more challenging, e.g. lung adenocarcinomas that are characterized by low numbers of cancer cells in the available cytological or histological samples.

## Supporting information

S1 Fig Example showing how to set the threshold when analyzing SensiScreen^®^ real-time PCR data. The threshold is set at 10% of the normalized fluorescence signal of the reference assay at cycle 45 (black line). The threshold is subsequently used to read the Ct of the ref and the mutation-specific assay (mut). The difference in threshold cycle DCt is calculated by subtracting the Ct of the reference assay from the Ct of the mutation-specific assay. Ct, threshold cycle; ref, reference assay; mut, mutation-specific assay; DCt, difference in threshold cycle.
(PDF)

S2 Fig MyGo Pro real-time PCR amplification plots of SensiScreen^®^ *KRAS* exon 2 simplex assays using serial dilutions of mutated DNA in a wild type background. 50 ng and/or approximately 16,000 copies of DNA was added to each reaction. The threshold was set at 10% of the average fluorescence signal of the reference assay at cycle 45. Legend describes the fraction of cell line DNA and/or mutated copies of *KRAS* exon 2 templates.
(PDF)

S3 Fig MyGo Pro real-time PCR amplification plots of SensiScreen^®^ *KRAS* exon 2 multiplex assays using serial dilutions of mutated DNA in a wild type background. 50 ng and/or approximately 16,000 copies of DNA was added to each reaction. The threshold was set at 10% of the average fluorescence signal of the reference assay at cycle 45. Legend describes the fraction of cell line DNA and/or mutated copies of *KRAS* exon 2 templates.
(PDF)

S1 Table Sequences of oligonucleotides used for construction of model templates for DS, ME-PCR and SensiScreen^®^ assays. Proprietary modifications not shown. DS, direct sequencing; ME-PCR, mutant-enriched PCR.
(PDF)

S2 Table Cell lines used for sensitivity studies. *kindly provided by Fondazione IRCCS Istituto Nazionale dei Tumori, Milan, Italy.
(PDF)

S3 Table Clinical-pathological patient data of cohort 1 used for analysis by direct sequencing, mutant-enriched PCR and SensiScreen^®^ simplex and multiplex. TNM, classification of malignant tumours (T, tumor; N, lymph nodes; M, metastasis); F, female; M, male.
(PDF)

S4 Table Clinical-pathological patient data of cohort 2 used for analysis by direct sequencing, therascreen^®^ *KRAS* test and SensiScreen^®^ simplex (patient 1–79). TNM, classification of malignant tumours (T, tumor; N, lymph nodes; M, metastasis); F, female; M, male.
(PDF)

S5 Table Clinical-pathological patient data of cohort 3 used for analysis by cobas^®^ *KRAS* mutation test and SensiScreen^®^ multiplex. TNM, classification of malignant tumours (T, tumor; N, lymph nodes; M, metastasis); F, female; M, male.
(PDF)

S6 Table *KRAS* exon 2 codon 12/13 mutational status of patients in cohort 1 determined by DS, mutant-enriched PCR ME-PCR and SensiScreen^®^ simplex and multiplex. Mutated cases found only by SensiScreen^®^ are underlined (sample 99) while cases found both by SensiScreen^®^ and ME-PCR are in bold (n = 15). *As determined by both SensiScreen^®^ simplex and multiplex. n, number; DS, direct sequencing; ME-PCR, mutant-enriched PCR; NE, not evaluable.
(PDF)

S7 Table *KRAS* exon 2 codon 12/13 mutational status of patients in cohort 2 determined by DS, therascreen^®^ and SensiScreen^®^ simplex and multiplex. Mutated cases found both by SensiScreen^®^ and therascreen^®^ are in bold (sample 59+70). n, number; DS, direct sequencing; WT, wild- type; NE: not evaluable.
(PDF)

S8 Table *KRAS* exon 2 codon 12/13 mutational status of patients in cohort 3 determined by cobas^®^ and SensiScreen^®^ simplex and multiplex. Mutated cases identified by SensiScreen^®^ but not by cobas^®^ are underlined (sample 5, 53, 131, 170, 189, and 214). n, number; WT, wild- type.
(PDF)
