# Supplementary figures and images for: SensiScreen® KRAS exon 2-sensitive simplex and multiplex real-time PCR-based assays for detection of KRAS exon 2 mutations
Source: PLoS One. 2017 Jun 21;12(6):e0178027. doi: 10.1371/journal.pone.0178027 (PMC5479524; doi:10.1371/journal.pone.0178027)

S1 Fig

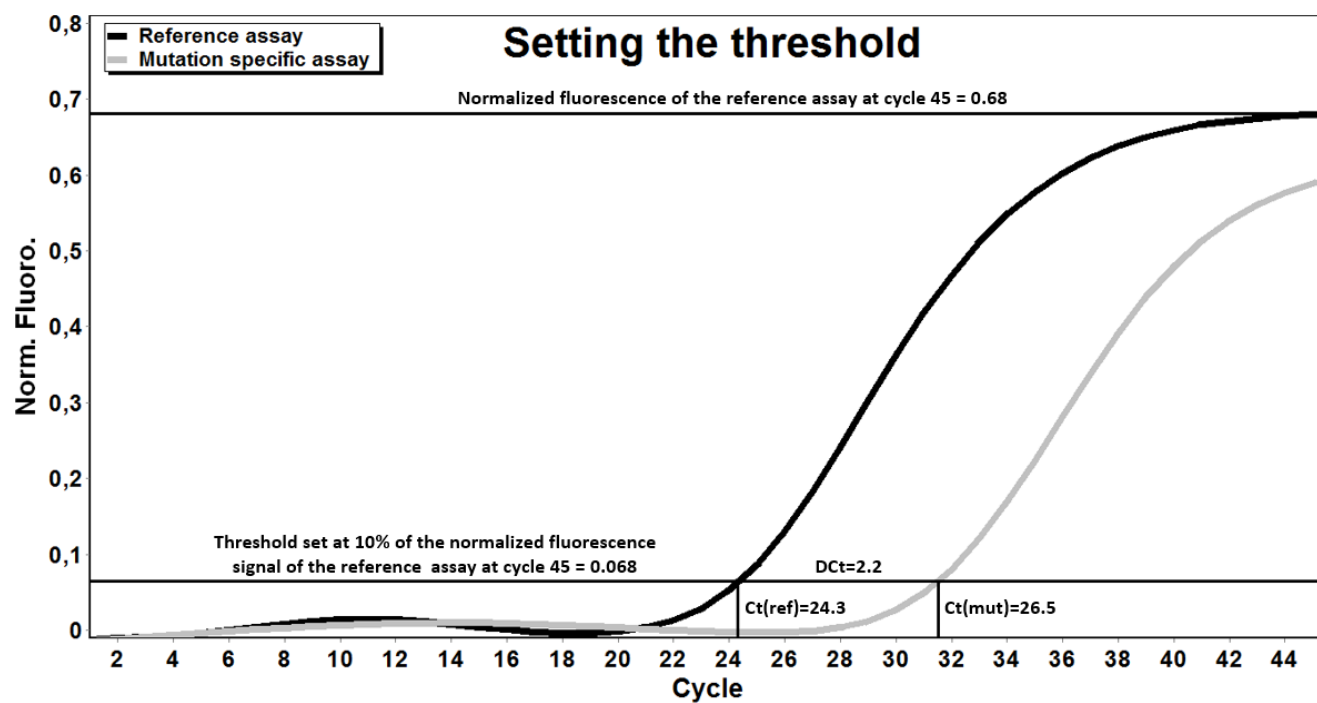

Supplement: S1 Fig — The threshold is set at 10% of the normalized fluorescence signal of the reference assay at cycle 45 (black line). The threshold is subsequently used to read the Ct of the ref and the mutation-specific assay (mut). The difference in threshold cycle DCt is calculated by subtracting the Ct of the reference assay from the Ct of the mutation-specific assay. Ct, threshold cycle; ref, reference assay; mut, mutation-specific assay; DCt, difference in threshold cycle. (PDF) [file pone.0178027.s001.pdf]

S2 Fig

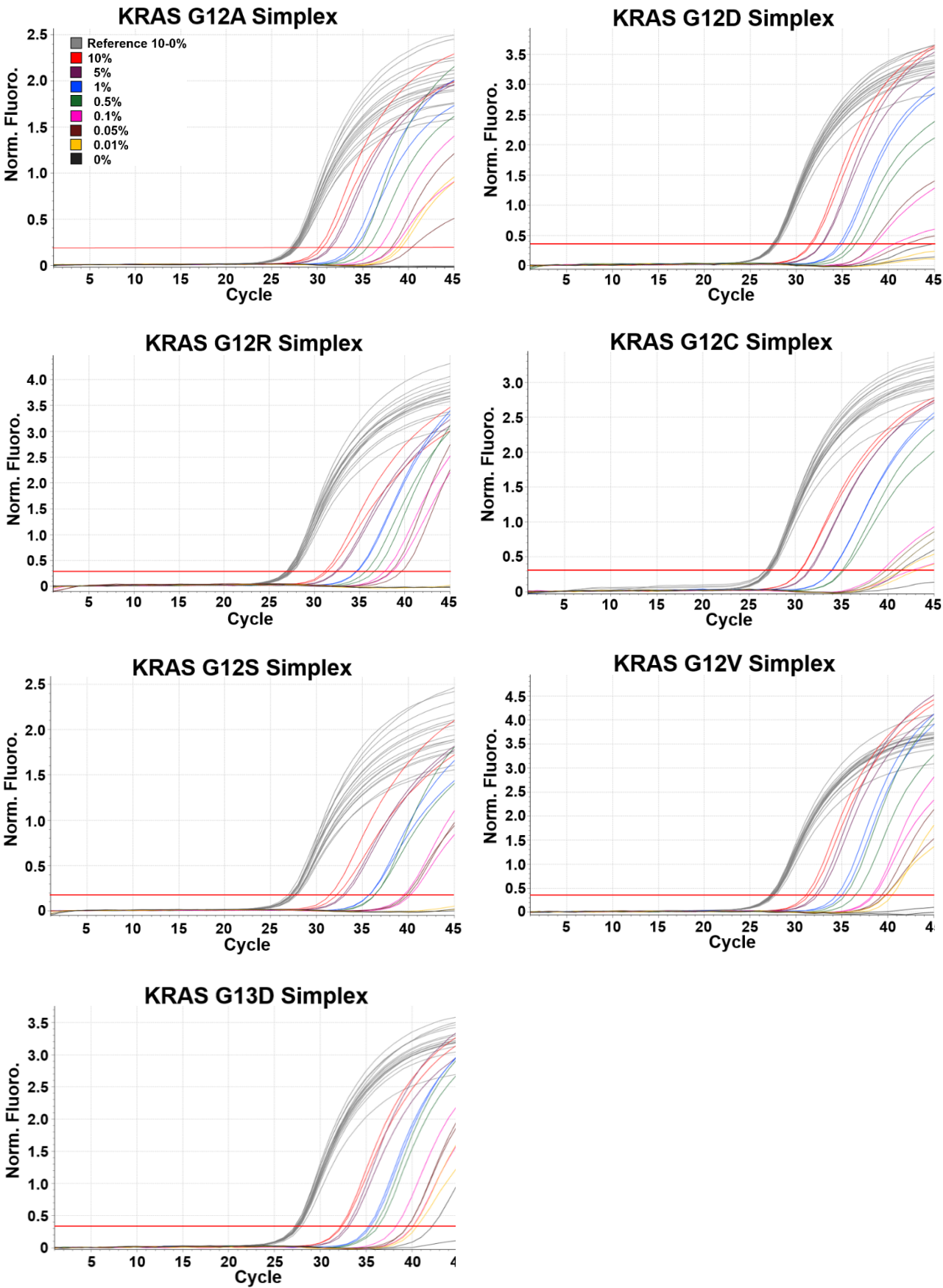

Supplement: S2 Fig — 50 ng and/or approximately 16,000 copies of DNA was added to each reaction. The threshold was set at 10% of the average fluorescence signal of the reference assay at cycle 45. Legend describes the fraction of cell line DNA and/or mutated copies of KRAS exon 2 templates. (PDF) [file pone.0178027.s002.pdf]

S3 Fig

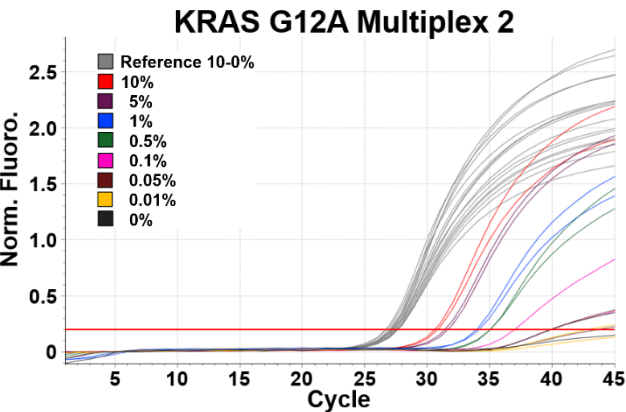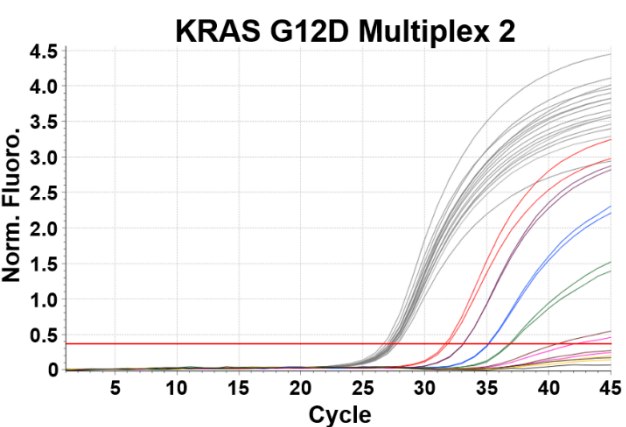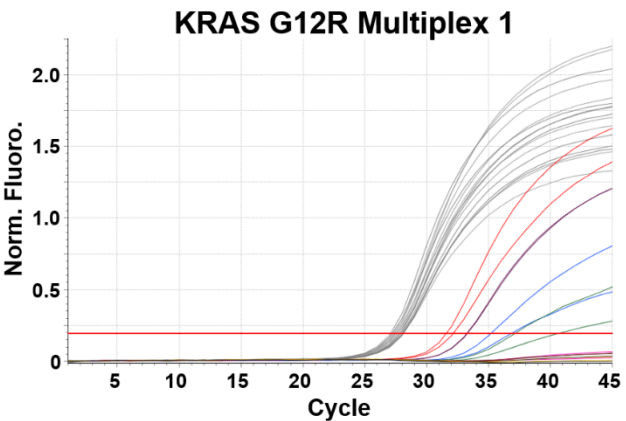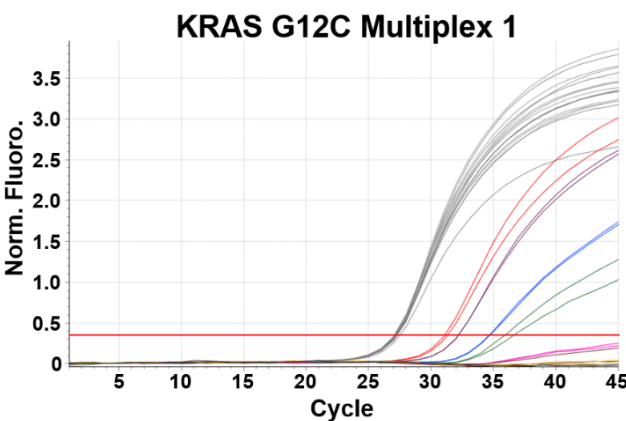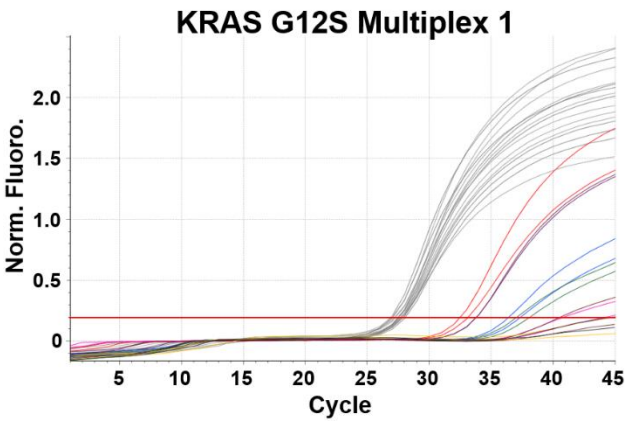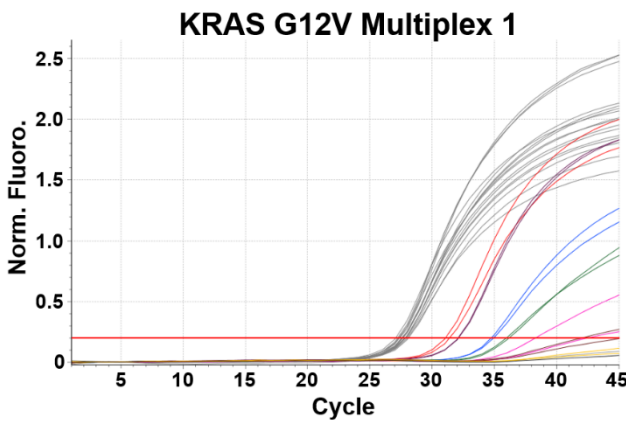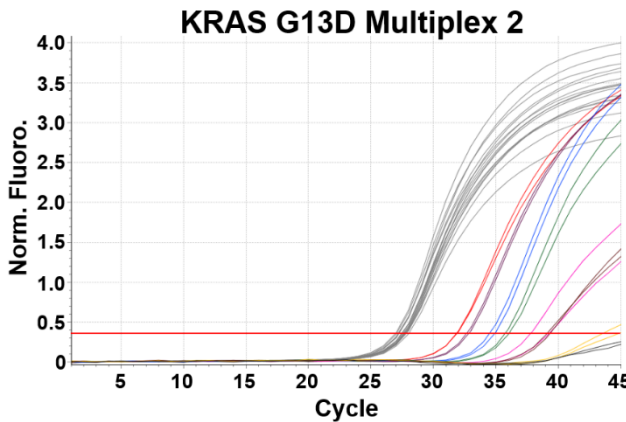

Supplement: S3 Fig — 50 ng and/or approximately 16,000 copies of DNA was added to each reaction. The threshold was set at 10% of the average fluorescence signal of the reference assay at cycle 45. Legend describes the fraction of cell line DNA and/or mutated copies of KRAS exon 2 templates. (PDF) [file pone.0178027.s003.pdf]
