# Supplementary material for: SensiScreen® KRAS exon 2-sensitive simplex and multiplex real-time PCR-based assays for detection of KRAS exon 2 mutations
Source: PLoS One. 2017 Jun 21;12(6):e0178027. doi: 10.1371/journal.pone.0178027 (PMC5479524; doi:10.1371/journal.pone.0178027)
Supplement: S1 Table — Proprietary modifications not shown. DS, direct sequencing; ME-PCR, mutant-enriched PCR. (PDF) [file pone.0178027.s004.pdf]

S1 Table

Primer name	Primer sequence 5´-> 3´
KRAS cloning forward	*AAAGGTACTGGTGGAGTATTTGATAG*
KRAS cloning reverse	*ACAAGATTTACCTCTATTGTTGGATC*
DS forward	*TGGTGGAGTATTTGATAGTGTA*
DS reverse	*CATGAAAATGGTCAGAGAA*
ME-PCR forward (1. PCR)	*ACTGAATATAAACTTGTGGTAGTTGGACCT*
ME-PCR reverse (1. PCR)	*ACTCATGAAAATGGTCAGAGAAACCTTTAT*
ME-PCR forward (2. PCR)	*ACTGAATATAAACTTGTGGTAGTTGGACCT*
ME-PCR reverse (2. PCR)	*TCAAAGAATGGTCCTGGACC*
SensiScreen® G12A forward	*AAACTTGTGGTAGTTGGAGCTTC*
SensiScreen® G12D forward	*AAACTTGTGGTAGTTGGAGCCGA*
SensiScreen® G12R forward	*AACTTGTGGTAGTTGGAGTTC*
SensiScreen® G12C forward	*AAACTTGTGGTAGTTGGAGCCT*
SensiScreen® G12S forward	*AAACTTGTGGTAGTTGGAGGTA*
SensiScreen® G12V forward	*ACTTGTGGTAGTTGGAGCCGT*
SensiScreen® G13D forward	*GTGGTAGTTGGAGCTGGAGA*
SensiScreen® reverse	*TACCTCTATTGTTGGATCATATTCGT*
SensiScreen® probe	*Penta Green™-AGCTGTATCGTCAAGGCACTC-Green Quencher*
SensiScreen® blocker	*AGCTGGTGGCGTAGG*
SensiScreen internal control probe	*Penta Yellow™-CTACTCCACTGCTGTCTAT-Yellow Quencher*
SensiScreen internal control forward	*CCCTAGAGTTGCCACAGC*
SensiScreen internal control reverse	*GGTAAGCAGCAAGAGAGC*
