# Supplementary material for: SensiScreen® KRAS exon 2-sensitive simplex and multiplex real-time PCR-based assays for detection of KRAS exon 2 mutations
Source: PLoS One. 2017 Jun 21;12(6):e0178027. doi: 10.1371/journal.pone.0178027 (PMC5479524; doi:10.1371/journal.pone.0178027)
Supplement: S2 Table — *kindly provided by Fondazione IRCCS Istituto Nazionale dei Tumori, Milan, Italy. (PDF) [file pone.0178027.s005.pdf]

S2 Table

**Cell line**	**Mutation**	**Zygosity**	**Origin**
SW1116 (ATCC® CCL-233™)	Gly12Arg (G12A, *c.35G>C*)	Homozygous	Colorectal cancer
MICOL29*	Gly12Asp (G12D, c.*35G>A*)	Homozygous	Colorectal cancer
CAL-62 (DSMZ®, ACC-448™)	Gly12Arg (G12R, *c.34G>C*)	Homozygous	Thyroid cancer
A549 (ATCC® CCL-185™)	Gly12Ser (G12S, *c.34G>A*)	Homozygous	Lung cancer
SW480 (ATCC® CCL-228™)	Gly12Val (G12V, *c.35G>T*)	Homozygous	Colorectal cancer
T84 (ATCC® CCL-248™)	Gly13Asp (G13D,*c.38G>A*)	Heterozygous	Colorectal cancer
