# Supplementary material for: SensiScreen® KRAS exon 2-sensitive simplex and multiplex real-time PCR-based assays for detection of KRAS exon 2 mutations
Source: PLoS One. 2017 Jun 21;12(6):e0178027. doi: 10.1371/journal.pone.0178027 (PMC5479524; doi:10.1371/journal.pone.0178027)
Supplement: S3 Table — TNM, classification of malignant tumours (T, tumor; N, lymph nodes; M, metastasis); F, female; M, male. (PDF) [file pone.0178027.s006.pdf]

S3 Table

Patient	Sex	Age	Localization	TNM
1	M	56	sigmoid colon	pT3/pN0
2	M	80	sigmoid colon	pT3/pN0
3	F	69	sigmoid colon	pT1/pN0
4	M	67	sigmoid colon	pT3/pN0
5	M	93	sigmoid colon	pT3/pN0
6	F	78	rectum	pT2
7	M	56	cecum	pT4/pN1
8	M	74	cecum	pT4
9	M	61	sigmoid colon	pT3/pN1
10	M	71	sigmoid colon	pT3
11	M	69	hepatic flexure	pT4
12	F	91	cecum	pT3
13	M	75	sigmoid colon	pT4
14	M	83	transverse colon	pT3/pN0
15	M	76	ascending colon/cecum	pT3/pN2/pM1
16	F	81	cecum	pT3/pN0
17	F	75	cecum	pT4/pN2
18	M	71	descending colon/cecum	pT3
19	F	71	hepatic flexure	pT3/pN1
20	F	75	sigmoid colon	pT3/pN0
21	M	84	sigmoid colon	pT3
22	M	79	ascending colon/cecum	pT3/pN0
23	M	71	sigmoid colon	pT3/pN1
24	M	73	rectum	pT3/pN0
25	M	61	sigmoid colon	pT3/pN2
26	F	90	rectum	pT3/pN1
27	M	80	cecum	pT3/pN0
28	F	91	sigmoid colon	pT3/pN1
29	F	83	sigmoid colon	pT3/pN2
30	F	81	sigmoid colon / rectum	pT4/pN1
31	M	62	sigmoid colon	pT4
32	M	81	rectum	pT3
33	F	96	sigmoid colon	pT4
34	F	59	transverse colon	pT4/pN0
35	M	76	ascending colon/cecum	pT3/pN1
36	M	77	cecum	pT3/pN0
37	F	73	ascending colon	pT3/pN2
38	F	73	sigmoid colon	pT4
39	F	81	ascending colon	pT2/pN0
40	F	46	ascending colon	pT2/pN0
41	M	79	descending colon	pT3
42	F	80	hepatic flexure	pT2/pN1
43	F	70	ascending colon	pT4/pN1
44	F	66	descending colon	pT3
45	F	64	cecum	pT3
46	F	69	transverse colon	pT3/pN0
47	M	84	rectum	pT2/pN0
48	M	49	ascending colon	pT3/pN2
49	M	60	sigmoid colon	pT3
50	M	71	rectum	pT4/pN0
51	M	64	ascending colon	pT3/pN1

52	F	70	ascending colon	pT3
53	M	70	ascending colon	pT3/pN0
54	F	65	sigmoid colon	pT2/pN0
55	F	77	ascending colon	pT3/pN0
56	F	81	sigmoid colon	pT4/pN0
57	F	72	sigmoid colon	pT3
58	M	83	ascending colon	pT3
59	F	87	descending colon	pT4/pN1
60	M	77	rectum	pT3/pN1
61	F	80	cecum	pT3
62	M	76	hepatic flexure	pT3/pN2
63	M	78	cecum	pT3
64	M	77	sigmoid colon	pT1
65	M	76	splenic flexure	pT4/pN1
66	F	59	descending colon	pT1
67	M	61	ascending colon	pT4
68	M	88	sigmoid colon	pT3
69	F	78	ascending colon	pT2
70	M	47	hepatic flexure	pT3/pN0
71	F	78	cecum	pT3
72	F	60	ascending colon	pT4/pN0
73	M	50	sigmoid colon / rectum	pT4/pN2
74	F	71	cecum	pT3/pN1
75	M	74	hepatic flexure	pT2
76	F	93	cecum	pT4/pN1
77	M	65	transverse colon	pT3/pN0
78	F	90	cecum	pT4/pN0
79	M	60	sigmoid colon	pT2
80	M	77	cecum	pT4
81	M	78	cecum	pT3
82	F	78	sigmoid colon	pT3/pN1
83	M	42	descending colon	pT3/pN0
84	F	65	hepatic flexure	pT3/pN1
85	F	62	sigmoid colon / rectum	pT3
86	F	61	cecum	pT2/pN0
87	M	85	hepatic flexure	pT3/pN0
88	F	78	descending colon	pT4/pN2
89	F	87	descending colon	pT4/pN0
90	F	80	sigmoid colon	pT3/pN0
91	F	84	cecum	pT3/pN1
92	M	73	descending colon	pT3/pN0
93	F	54	ascending colon	pT2/pN0
94	M	82	hepatic flexure	pT3/pN0
95	M	64	cecum	pT3/pN2
96	M	88	splenic flexure	pT3/pN0
97	F	69	transverse colon	pT4
98	F	85	cecum	pT3/pN0
99	F	85	ascending colon	pT2/pN0
100	F	72	rectum	pT2/pN0
