# Supplementary material for: SensiScreen® KRAS exon 2-sensitive simplex and multiplex real-time PCR-based assays for detection of KRAS exon 2 mutations
Source: PLoS One. 2017 Jun 21;12(6):e0178027. doi: 10.1371/journal.pone.0178027 (PMC5479524; doi:10.1371/journal.pone.0178027)
Supplement: S4 Table — TNM, classification of malignant tumours (T, tumor; N, lymph nodes; M, metastasis); F, female; M, male. (PDF) [file pone.0178027.s007.pdf]

S4 Table

Patient	Sex	Age	Localization	TNM
1	M	73	biopsy (sigmoid colon)	ND
2	F	68	ascending colon	ND
3	F	78	ascending colon	pT3/pN0
4	F	81	biopsy (rectum)	ND
5	M	84	biopsy (hepatic flexure)	ND
6	M	70	biopsy (transverse colon)	ND
7	F	79	ascending colon	ND
8	F	68	Unkown	ND
9	F	81	sigmoid colon	pT3/pN1
10	F	74	biopsy (sigmoid colon)	ND
11	F	63	biopsy (sigmoid colon)	ND
12	M	72	biopsy (hepatic flexure)	ND
13	M	76	rectum	pT3/pN0
14	M	79	rectum	pT3/pN2
15	F	78	lung (rectum)	ND
16	M	70	sigmoid colon	ND
17	M	83	biopsy (sigmoid colon)	ND
18	M	79	sigmoid colon	ND
19	M	77	cecum	pT3/pN0
20	F	69	sigmoid colon	pT3/pN0
21	M	36	small intestine	ND
22	F	77	splenic flexure	pT3/pN1
23	M	51	biopsy (ascending colon)	ND
24	M	79	rectum (mucosa)	ND
25	F	81	splenic flexure	pT3/pN1
26	M	50	biopsy (ascending colon)	ND
27	M	70	sigmoid colon	ND
28	M	63	rectum	pT2/pN0
29	F	59	sigmoid colon	pT4/pN2
30	M	64	liver (rectum)	ND
31	M	70	ascending colon	pT3/pN1/pM9
32	F	55	cecum	pT2/pN2
33	F	70	sigmoid colon	pT3/pN2
34	F	70	liver (sigmoid colon)	ND
35	F	64	pleura (rectum)	ND
36	M	67	rectum	pT3/pN1
37	M	60	ascending colon	pT4/pN0
38	F	67	cecum	pT4/pN2
39	M	81	biopsy (sigmoid colon)	ND
40	F	87	cecum	pT4/pN2
41	M	71	biopsy (sigmoid colon)	ND
42	M	85	ascending colon	pT3/pN2
43	F	72	liver (colon)	ND
44	M	56	rectum	pT3/pN0
45	M	71	liver (descending colon)	ND
46	M	77	rectum	pT3/pN1
47	M	56	liver (rectum)	ND
48	F	78	ascending colon	pT4/pN2
49	M	61	descending colon	pT4/pN0

50	F	53	cecum	pT4/pN2
51	M	76	transverse colon	pT3/pN1/pM0
52	M	75	biopsy (rectum)	ND
53	M	75	abdominal wall (sigmiod colon)	ND
54	M	75	sigmoid colon	pT3/pN0/pM0
55	M	62	biopsy (rectum)	ND
56	M	71	sigmoid colon	pT1/pN1
57	F	35	descending colon	pT4/pN2/pM1
58	F	69	ascending colon	pT3/pN2/pM9
59	F	72	liver (rectum)	ND
60	M	60	rectum	pT4/pN2/pM0
61	M	58	sigmoid colon	pT3/pN2/pM1
62	M	86	rectum (mucosa)	ND
63	M	63	rectum	pT3/pN1
64	F	78	rectum	pT3/pN1
65	M	73	sigmoid colon	pT4/pN0
66	M	52	biopsy (sigmoid colon)	ND
67	M	88	liver (rectum)	ND
68	F	68	descending colon	ND
69	F	65	sigmoid colon	pT3/pN2
70	F	82	biopsy (rectum)	ND
71	M	68	sigmoid colon (mucosa)	ND
72	F	57	biopsy (rectum)	ND
73	M	82	biopsy (rectum)	ND
74	M	75	acites fluid	ND
75	M	71	rectum	pT3/pN2/pM9
76	M	82	biopsy (ascending colon)	ND
77	M	53	liver (rectum)	ND
78	M	77	lung (rectum)	ND
79	F	75	liver (rectum)	ND
