# Supplementary material for: SensiScreen® KRAS exon 2-sensitive simplex and multiplex real-time PCR-based assays for detection of KRAS exon 2 mutations
Source: PLoS One. 2017 Jun 21;12(6):e0178027. doi: 10.1371/journal.pone.0178027 (PMC5479524; doi:10.1371/journal.pone.0178027)
Supplement: S5 Table — TNM, classification of malignant tumours (T, tumor; N, lymph nodes; M, metastasis); F, female; M, male. (PDF) [file pone.0178027.s008.pdf]

S5 Table

Patient	Sex	Age	Localization	TNM
1	F	48	rectum	pT4/pN0
2	M	84	sigmoid colon	pT3/pN2
3	F	75	sigmoid colon	pT3/pN0
4	F	54	sigmoid colon	pT3/pN1
5	M	86	rectum	pT3/pN2
6	F	51	splenic flexure	pT3/pN0
7	F	81	transverse colon	pT4/pN2
8	M	81	rectum	pT3/pN0
9	M	82	rectum	pT3/pN2
10	M	30	transverse colon	pT3/pN0
11	F	54	rectum	pT3/pN1
12	M	71	transvere colon	pT3/pN0
13	F	51	sigmoid colon	pT4/pN1
14	M	88	hepatic flexure	ND
15	F	76	sigmoid colon	pT2/pN0
16	F	63	cecum	pT3/pN0
17	M	52	rectum	pT3/pN0
18	M	72	polyp (sigmoid colon)	ND
19	M	68	rectum (mucosa)	ND
20	F	64	sigmoid colon	pT4/pN2
21	F	59	rectum	pT3/pN0
22	F	74	ascending colon	pT3/pN0
23	F	70	polyp (sigmoid colon)	pT1/ND
24	F	66	sigmoid colon	pT2/pN0
25	M	82	rectum	pT2/pN1
26	F	78	ascending colon	pT3/pN0
27	M	71	polyp (cecum)	ND
28	M	71	descending colon	pT3/pN1
29	M	63	cecum	pT3/pN0
30	M	48	cecum	pT4/pN2
31	M	70	polyp (sigmoid colon)	ND
32	F	69	rectum	pT3/pN0
33	F	57	sigmoid colon	pT3/pN2
34	M	64	sigmoid colon	pT4/pN1
35	F	64	transverse colon	pT3/pN0
36	M	77	polyp (rectum)	pT1/ND
37	M	81	rectum (moucosa)	ND
38	F	80	mamma (colon)	ND
39	M	80	rectum	pT3/pN0
40	M	66	rectum	pT3/pN0
41	M	92	ascending colon	pT3/pN0
42	F	82	hepatic flexure	pT1/pN0
43	F	75	transverse colon	pT4/pN0
44	M	78	sigmoid colon	pT3/pN2
45	F	87	ascending colon	pT2/pN0
46	M	55	polyp (colon/rectum)	ND
47	M	58	liver (colon cancer)	ND
48	F	56	rectum (mucosa)	ND
49	F	85	sigmoid colon	pT4/pN2

50	M	67	rectum	pT3/pN0
51	M	60	hepatic flexure (mucosa)	ND
52	M	49	rectum	pT3/pN0
53	M	78	rectum	pT2/pN0
54	M	82	rectum	pT3/pN0
55	M	73	transverse colon	pT3/pN1
56	M	79	rectum	pT1/pN2
57	F	79	ascending colon	pT1/pN0
58	M	70	descending colon	pT4/pN0
59	F	75	sigmoid colon	pT3/pN0
60	F	68	transverse colon	pT3/pN0
61	M	56	descending colon	pT3/pN0
62	F	93	hepatic flexure	pT3/pN0
63	F	66	polyp (rectum)	ND
64	F	75	cecum	pT4/pN0
65	F	89	cecum	pT3/pN0
66	M	64	rectum	pT3/pN1
67	F	68	sigmoid colon	pT3/pN2
68	M	76	sigmoid colon	pT4/pN2
69	M	92	hepatic flexure	pT3/pN1
70	F	87	transverse colon	pT3/pN1
71	F	76	transverse colon	pT3/pN2
72	M	77	biopsy (rectum)	ND
73	F	65	rectum	pT3/ND
74	F	77	descending colon	pT3/pN1
75	M	82	biopsy (rectum)	ND
76	F	63	biopsy (ascending colon)	ND
77	F	79	rectum	pT2/pN0
78	M	77	hepatic flexure	pT3/pN0
79	M	71	hepatic flexure	pT3/pN1
80	M	78	rectum	pT1/pN0
81	M	63	rectum	pT2/pN0
82	F	60	biopsy (cecum)	ND
83	M	73	biopsy (sigmoid colon)	ND
84	M	70	sigmoid colon	pT2/pN2
85	M	50	transverse colon	pT4/pN1
86	F	56	polyp (ascending colon)	ND
87	F	63	sigmoid colon	pT3/pN0
88	M	78	sigmoid colon	pT4/pN1
89	M	85	polyp (hepatic flexure)	ND
90	M	66	biopsy (mucosa colon)	ND
91	M	76	rectum	pT3/pN0
92	M	74	biopsy (ascending colon)	ND
93	M	69	polyp (sigmoid colon)	ND
94	M	73	rectum	pT2/pN0
95	M	57	sigmoid colon	pT3/pN1
96	M	76	cecum	pT3/pN2
97	F	82	rectum	pT4/pN1
98	M	64	rectum	pT2/pN0
99	M	69	transverse colon	pT3/pN0
100	M	77	rectum	pT3/pN1
101	M	87	liver (rectum)	ND

102	M	76	ascending colon	pT3/pN0
103	F	71	descending colon	pT4/pN1
104	F	87	rectum	pT3/pN1
105	F	67	rectum	pT2/pN0
106	M	63	rectum	pT4/pN2
107	M	73	sigmoid colon	pT3/pN0
108	M	74	sigmoid colon	pT4/pN2
109	M	82	transverse colon	pT4/pN0
110	F	62	rectum	pT3/pN0
111	M	72	rectum	pT3/pN2
112	F	78	sigmoid colon	pT4/pN2
113	F	53	sigmoid colon	pT3/pN0
114	M	82	biopsy (rectum)	ND
115	F	81	sigmoid colon	pT3/pN1
116	F	84	polyp (ascending colon)	pT1/pN0
117	F	90	polyp (rectum/sigmoid colon)	pT2/pN0
118	F	54	transverse colon	pT3/pN1
119	F	64	polyp (rectum)	ND
120	F	74	sigmoid colon	pT2/pN0
121	F	51	rectum	pT3/pN1
122	M	79	rectum	pT4/pN2
123	F	85	polyp (sigmoid colon)	ND
124	M	92	rectum	pT1/ND
125	M	72	polyp (sigmoid colon)	ND
126	F	72	polyp (sigmoid colon)	ND
127	F	75	rectum	pT1/pN0
128	F	56	splenic flexure	pT4/pN2
129	M	74	rectum	pT3/pN1
130	F	84	hepatic flexure	pT3/pN1
131	M	68	urine bladder (rectum)	ND
132	M	31	rectum	pT3/pN0
133	M	59	rectum	pT3/pN1
134	M	71	rectum	pT3/pN0
135	M	66	rectum	pT3/pN1
136	M	84	sigmoid colon	pT2/pN0
137	M	72	rectum	pT3/pN1
138	F	80	liver (rectum)	ND
139	M	60	rectum	pT3/pN0
140	M	71	rectum	pT3/pN0
141	M	68	urine bladder (rectum)	ND
142	M	68	ascending colon	pT4/pN2
143	M	55	biopsy (rectum mucosa)	ND
144	M	76	transverse colon	pT3/pN1
145	M	76	polyp (sigmoid colon)	ND
146	M	67	hepatic flexure	pT2/pN0
147	M	69	sigmoid colon	pT4/pN0
148	F	79	rectum	pT3/pN0
149	F	90	transverse colon	pT4/pN0
150	M	80	rectum	pT2/pN0
151	F	71	transverse colon	pT3/pN0
152	M	81	sigmoid colon	pT1/pN0
153	M	63	rectum	pT3/pN0

154	M	66	sigmoid colon	pT3/pN0
155	F	71	cecum	pT3/pN0
156	F	59	sigmoid colon	pT2/pN0
157	F	69	cecum	pT3/pN0
158	F	78	sigmoid colon	pT2/pN0
159	M	57	sigmoid colon	pT3/pN2
160	M	63	rectum	pT2/pN0
161	F	73	biopsy (rectum)	ND
162	F	71	cecum	pT4/pN2/pM1
163	F	69	polyp (ascending colon)	ND
164	F	63	sigmoid colon	pT3/pN1
165	M	83	ascending colon	pT3/pN0
166	M	76	sigmoid colon	pT3/pN2
167	F	71	sigmoid colon	pT2/pN1
168	M	78	hepatic flexure	pT3/pN0
169	F	86	sigmoid colon	pT4/pN0
170	M	72	rectum	pT3/pN1
171	M	74	rectum	pT3/pN0
172	M	65	liver (rectum)	ND
173	M	65	liver (rectum)	ND
174	M	68	sigmoid colon	pT2/pN0/pM0
175	F	74	ascending colon	pT2/pN0
176	M	28	splenic flexure	pT4/pN2
177	M	62	rectum	pT3/pN0
178	F	74	rectum	pT3/pN2
179	M	86	sigmoid colon	pT4/pN0
180	F	96	sigmoid colon	pT4/pN2
181	M	46	rectum	pT4/pN0
182	M	80	rectum	pT4/pN2/pM1
183	M	58	rectum	pT2/pN0
184	F	68	descending colon	pT4/pN0
185	M	67	rectum	pT3/pN2
186	M	87	descending colon	pT2/pN0
187	F	65	rectum	pT3/pN0
188	M	71	rectum	pT3/pN0
189	M	66	polyp (ascending colon)	ND
190	M	77	ascending colon	pT4/pN1
191	F	74	ascending colon	pT3/pN2
192	M	82	rectum	pT3/pN1
193	M	88	rectum	pT2/pN1
194	M	70	sigmoid colon	pT4/pN2
195	F	87	ascending colon	pT2/pN0
196	F	65	biopsy (rectum)	ND
197	M	80	descending colon	pT4/pN1
198	M	61	descending colon	pT3/pN0
199	M	61	transvere colon	pT3/pN0
200	M	79	sigmoid colon	pT3/pN0
201	M	72	sigmoid colon	pT3/pN2
202	M	68	rectum	pT3/pN0
203	M	84	ascending colon	pT3/pN1
204	F	77	descending colon	pT3/pN0
205	F	82	ascending colon	pT4/pN2

206	M	73	cecum	pT4/pN1/pM1
207	F	80	rectum	pT3/pN0
208	M	62	ascending colon	pT3/pN1
209	F	74	cecum	pT3/pN0
210	M	64	descending colon	pT3/pN0
211	M	62	transvere colon	pT4/pN1
212	M	82	rectum	pT2/pN0
213	F	77	cecum	pT4/pN0
214	F	82	descending colon	pT2/pN1
215	M	62	polyp (rectum)	ND
216	M	52	polyp (rectum)	ND
217	F	70	descending colon	pT4/pN2
218	F	62	ascending colon	pT3/pN2
219	M	74	rectum	pT1/pN0
220	M	66	splenic flexure	pT3/pN2
221	F	68	sigmoid colon	pT3/pN2
222	M	72	biopsy (rectum/sigmoid colon)	ND
223	M	69	descending colon	pT3/pN0
224	M	71	biopsy (rectum)	ND
225	M	77	ascending colon	pT3/pN2
226	M	71	sigmoid colon	pT1/pN0
227	M	62	rectum	pT3/pN2
228	M	80	transvere colon	pT4/pN0
229	M	75	sigmoid colon	pT1/pN0
230	F	82	cecum	pT3/pN0
231	F	68	rectum	pT3/pN0
232	M	76	sigmoid colon	pT4/pN1
233	F	65	rectum/sigmoid colon	ND
234	M	77	ascending colon	pT3/pN0
235	F	74	rectum	pT3/pN2
236	M	69	sigmoid colon	pT2/pN0
237	F	67	rectum	pT3/pN0
238	M	75	cecum	pT2/pN0
239	F	76	ascending colon	pT3/pN0
240	M	83	ascending colon	pT3/pN1
241	F	73	brain (colon cancer)	ND
242	F	90	hepatic flexure	pT3/pN0
243	M	77	ascending colon	pT3/pN0
244	M	76	sigmoid colon	pT4/pN1
245	F	87	sigmoid colon	pT3/pN0
246	M	76	transvere colon	pT4/pN0
247	M	80	rectum (recidive)	ND
248	M	64	descending colon	pT3/pN2
249	M	82	rectum	pT3/pN1
250	M	55	rectum	pT3/pN2
251	M	83	sigmoid colon	pT4/pN1
252	F	61	polyp (rectum)	ND
253	M	88	sigmoid colon	pT2/pN0
254	M	64	ascending colon	pT2/pN0
255	F	69	transvere colon	pT4/pN2
256	M	67	ascending colon	pT3/pN2
257	F	82	sigmoid colon	pT3/pN0

258	F	46	rectum	pT3/pN1
259	M	70	rectum	pT3/pN1
260	F	72	rectum / sigmoid colon	pT4/pN2
261	M	74	rectum	pT3/pN0
262	M	74	small intestine	ND
263	F	68	descending colon	pT3/pN0
264	M	83	cecum	pT2/pN0
265	M	75	ascending colon	pT2/pN0
266	M	61	rectum	pT3/pN1
267	M	69	descending colon	pT3/pN1
268	M	66	biposy (rectum)	ND
269	M	73	splenic flexure	pT4/pN2
270	F	72	hepatic flexure	pT2/pN0
271	M	82	rectum	pT3/pN0
272	F	68	rectum	pT4/pN0
273	M	80	rectum/sigmoid colon	pT4/pN0
274	M	80	descending colon	pT3/pN0
275	M	81	ascending colon	pT3/pN1
276	M	77	ascending colon	pT3/pN1
277	F	69	sigmoid colon	pT2/pN0
278	M	63	sigmoid colon	pT3/pN2
279	M	71	cecum	pT3/pN1
280	M	79	sigmoid colon	pT3/pN0
281	M	68	rectum /sigmoid colon	pT4/pN2
282	F	66	rectum	pT4/pN1
283	M	52	sigmoid colon	pT2/pN1
