# Supplementary material for: SensiScreen® KRAS exon 2-sensitive simplex and multiplex real-time PCR-based assays for detection of KRAS exon 2 mutations
Source: PLoS One. 2017 Jun 21;12(6):e0178027. doi: 10.1371/journal.pone.0178027 (PMC5479524; doi:10.1371/journal.pone.0178027)
Supplement: S6 Table — Mutated cases found only by SensiScreen® are underlined (sample 99) while cases found both by SensiScreen® and ME-PCR are in bold (n = 15). *As determined by both SensiScreen® simplex and multiplex. n, number; DS, direct sequencing; ME-PCR, mutant-enriched PCR; NE, not evaluable. (PDF) [file pone.0178027.s009.pdf]

S6 Table

Cohort 1							
**n**	**DS**	**ME-PCR**	**SensiScreen®***	**n**	**DS**	**ME-PCR**	**SensiScreen®**
1	WT	WT	WT	51	G13D	G13D	G13D
2	WT	WT	WT	52	WT	WT	WT
3	WT	WT	WT	53	G12D	G12D	G12D
4	WT	WT	WT	54	WT	WT	WT
5	WT	G12C	G12C	55	WT	WT	WT
6	WT	WT	WT	56	G12D	G12D	G12D
7	WT	WT	WT	57	WT	WT	WT
8	WT	WT	WT	58	G12D	G12D	G12D
9	WT	WT	WT	59	WT	WT	WT
10	G12D	G12D	G12D	60	G13D	G13D	G13D
11	WT	WT	WT	61	G12D	G12D	G12D
12	WT	WT	WT	62	WT	WT	WT
13	G12A	G12A	G12A	63	WT	**G12C**	**G12C**
14	WT	WT	WT	64	WT	WT	WT
15	WT	WT	WT	65	G13D	G13D	G13D
16	WT	WT	WT	66	WT	WT	WT
17	WT	WT	WT	67	WT	WT	WT
18	WT	**G13D**	**G13D**	68	WT	**G12C**	**G12C**
19	WT	WT	WT	69	G12V	G12V	G12V
20	WT	WT	WT	70	G12D	G12D	G12D
21	G12V	G12V	G12V	71	WT	WT	WT
22	WT	WT	WT	72	WT	**G12V**	**G12V**
23	WT	WT	WT	73	WT	WT	WT
24	WT	WT	WT	74	WT	WT	WT
25	WT	WT	WT	75	WT	WT	WT
26	WT	WT	WT	76	WT	**G12C**	**G12C**
27	WT	**G12D**	**G12D**	77	WT	WT	WT
28	WT	**G12V**	**G12V**	78	WT	WT	WT
29	WT	WT	WT	79	WT	WT	WT
30	G12D	G12D	G12D	80	WT	WT	WT
31	G12V	G12V	G12V	81	WT	WT	WT
32	G12R	G12R	G12R	82	WT	**G12V**	**G12V**
33	G12S	G12S	G12S	83	WT	WT	WT
34	WT	WT	WT	84	G12C	G12C	G12C
35	WT	WT	WT	85	G12D	G12D	G12D
36	G12V	G12V	G12V	86	WT	**G12S**	**G12S**
37	WT	WT	WT	87	WT	**G12A**	**G12A**
38	WT	WT	WT	88	WT	**G12V**	**G12V**
39	WT	WT	WT	89	WT	**G13D**	**G13D**
40	WT	WT	WT	90	WT	WT	WT
41	WT	WT	WT	91	WT	WT	WT
42	G12D	G12D	G12D	92	G12D	G12D	G12D
43	G12C	G12C	G12C	93	WT	WT	WT
44	G12A	G12A	G12A	94	WT	WT	WT
45	G12V	G12V	G12V	95^	NE	NE	NE
46	WT	WT	WT	96	WT	WT	WT
47	G12V	G12V	G12V	97	WT	**G12V**	**G12V**
48	G12D	G12D	G12D	98	WT	WT	WT
49	G12V	G12V	G12V	99	WT	WT	G13D
50	G12D	G12D	G12D	100	WT	**G12V**	**G12V**
				**n mutated cases**	**28**	**43**	**44**
