# Supplementary material for: SensiScreen® KRAS exon 2-sensitive simplex and multiplex real-time PCR-based assays for detection of KRAS exon 2 mutations
Source: PLoS One. 2017 Jun 21;12(6):e0178027. doi: 10.1371/journal.pone.0178027 (PMC5479524; doi:10.1371/journal.pone.0178027)
Supplement: S7 Table — Mutated cases found both by SensiScreen® and therascreen® are in bold (sample 59+70). n, number; DS, direct sequencing; WT, wild- type; NE: not evaluable. (PDF) [file pone.0178027.s010.pdf]

S7 Table

Cohort 2							
**n**	**DS**	**therascreen®**	**SensiScreen®**	**n**	**DS**	**therascreen®**	**SensiScreen®**
1	WT	WT	WT	41	WT	WT	WT
2	WT	WT	WT	42	G12A	G12A	G12A
3	WT	WT	WT	43	WT	WT	WT
4	WT	WT	WT	44	G12C	G12C	G12C
5	WT	WT	WT	45	WT	WT	WT
6	WT	WT	WT	46	WT	WT	WT
7	WT	WT	WT	47	G13D	G13D	G13D
8	WT	WT	WT	48	G12S	G12S	G12S
9	WT	WT	WT	49	G12D	G12D	G12D
10	WT	WT	WT	50	G12D	G12D	G12D
11	WT	WT	WT	51	WT	WT	WT
12	WT	WT	WT	52	WT	WT	WT
13	WT	WT	WT	53	WT	WT	WT
14	WT	WT	WT	54	WT	WT	WT
15	WT	WT	WT	55	WT	WT	WT
16	WT	WT	WT	56	G13D	G13D	G13D
17	WT	WT	WT	57	WT	WT	WT
18	WT	WT	WT	58	G13D	G13D	G13D
19	WT	WT	WT	59	WT	**G12S**	**G12S**
20	WT	WT	WT	60	G12A	G12A	G12A
21	WT	WT	WT	61	WT	WT	WT
22	WT	WT	WT	62	WT	WT	WT
23	WT	WT	WT	63	G12V	G12V	G12V
24	WT	WT	WT	64	WT	WT	WT
25	WT	WT	WT	65	WT	WT	WT
26	WT	WT	WT	66	WT	WT	WT
27	WT	WT	WT	67	G13D	G13D	G13D
28	WT	WT	WT	68	WT	WT	WT
29	WT	WT	WT	69	G13D	G13D	G13D
30	WT	WT	WT	70	WT	**G12C**	**G12C**
31	WT	WT	WT	71	WT	WT	WT
32	WT	WT	WT	72	WT	WT	WT
33	WT	WT	WT	73	G12C	G12C	G12C
34	WT	WT	WT	74	G12V	G12V	G12V
35	WT	WT	WT	75	WT	WT	WT
36	G12D	G12D	G12D	76	WT	WT	WT
37	G12D	G12D	G12D	77	WT	WT	WT
38	G12V	G12V	G12V	78	WT	WT	WT
39	WT	WT	WT	79	WT	WT	WT
40	WT	WT	WT				
				**n mutated cases**	**17**	**19**	**19**
