# Supplementary material for: SensiScreen® KRAS exon 2-sensitive simplex and multiplex real-time PCR-based assays for detection of KRAS exon 2 mutations
Source: PLoS One. 2017 Jun 21;12(6):e0178027. doi: 10.1371/journal.pone.0178027 (PMC5479524; doi:10.1371/journal.pone.0178027)
Supplement: S8 Table — Mutated cases identified by SensiScreen® but not by cobas® are underlined (sample 5, 53, 131, 170, 189, and 214). n, number; WT, wild- type. (PDF) [file pone.0178027.s011.pdf]

S8 Table

Cohort 3								
**n**	**cobas®**	**SensiScreen®**	n	**cobas®**	**SensiScreen®**	**n**	**cobas®**	**SensiScreen®**
1	Mutant	Mutant	56	WT	WT	111	WT	WT
2	WT	WT	57	WT	WT	112	Mutant	Mutant
3	WT	WT	58	WT	WT	113	Mutant	Mutant
4	WT	WT	59	WT	WT	114	WT	WT
5	WT	Mutant	60	WT	WT	115	WT	WT
6	WT	WT	61	WT	WT	116	Mutant	Mutant
7	WT	WT	62	WT	WT	117	WT	WT
8	WT	WT	63	Mutant	Mutant	118	WT	WT
9	WT	WT	64	WT	WT	119	WT	WT
10	Mutant	Mutant	65	Mutant	Mutant	120	Mutant	Mutant
11	Mutant	Mutant	66	WT	WT	121	Mutant	Mutant
12	Mutant	Mutant	67	Mutant	Mutant	122	WT	WT
13	WT	WT	68	WT	WT	123	WT	WT
14	WT	WT	69	WT	WT	124	WT	WT
15	WT	WT	70	WT	WT	125	Mutant	Mutant
16	Mutant	Mutant	71	WT	WT	126	WT	WT
17	Mutant	Mutant	72	Mutant	Mutant	127	Mutant	Mutant
18	WT	WT	73	WT	WT	128	WT	WT
19	WT	WT	74	WT	WT	129	Mutant	Mutant
20	WT	WT	75	Mutant	Mutant	130	WT	WT
21	WT	WT	76	WT	WT	131	WT	Mutant
22	WT	WT	77	WT	WT	132	Mutant	Mutant
23	WT	WT	78	WT	WT	133	WT	WT
24	Mutant	Mutant	79	Mutant	Mutant	134	WT	WT
25	WT	WT	80	WT	WT	135	Mutant	Mutant
26	Mutant	Mutant	81	WT	WT	136	Mutant	Mutant
27	Mutant	Mutant	82	WT	WT	137	WT	WT
28	Mutant	Mutant	83	WT	WT	138	Mutant	Mutant
29	WT	WT	84	WT	WT	139	WT	WT
30	Mutant	Mutant	85	WT	WT	140	WT	WT
31	WT	WT	86	WT	WT	141	WT	WT
32	WT	WT	87	WT	WT	142	WT	WT
33	WT	WT	88	WT	WT	143	WT	WT
34	WT	WT	89	WT	WT	144	Mutant	Mutant
35	WT	WT	90	WT	WT	145	WT	WT
36	WT	WT	91	WT	WT	146	WT	WT
37	WT	WT	92	WT	WT	147	Mutant	Mutant
38	WT	WT	93	Mutant	Mutant	148	WT	WT
39	Mutant	Mutant	94	WT	WT	149	Mutant	Mutant
40	WT	WT	95	WT	WT	150	Mutant	Mutant
41	WT	WT	96	Mutant	Mutant	151	WT	WT
42	WT	WT	97	WT	WT	152	WT	WT
43	WT	WT	98	WT	WT	153	WT	WT
44	WT	WT	99	WT	WT	154	WT	WT
45	Mutant	Mutant	100	WT	WT	155	Mutant	Mutant
46	WT	WT	101	Mutant	Mutant	156	Mutant	Mutant
47	WT	WT	102	WT	WT	157	WT	WT
48	WT	WT	103	WT	WT	158	WT	WT
49	WT	WT	104	Mutant	Mutant	159	WT	WT
50	WT	WT	105	WT	WT	160	WT	WT
51	WT	WT	106	Mutant	Mutant	161	Mutant	Mutant
52	Mutant	Mutant	107	Mutant	Mutant	162	WT	WT
53	WT	Mutant	108	WT	WT	163	WT	WT
54	WT	WT	109	WT	WT	164	WT	WT
55	WT	WT	110	Mutant	Mutant	165	WT	WT

Cohort 3 continued

n	cobas®	SensiScreen®	n	cobas®	SensiScreen®	n	cobas®	SensiScreen®
166	WT	WT	214	WT	Mutant	262	Mutant	Mutant
167	Mutant	Mutant	215	WT	WT	263	Mutant	Mutant
168	WT	WT	216	WT	WT	264	Mutant	Mutant
169	WT	WT	217	WT	WT	265	WT	WT
170	WT	Mutant	218	WT	WT	266	WT	WT
171	Mutant	Mutant	219	Mutant	Mutant	267	WT	WT
172	Mutant	Mutant	220	Mutant	Mutant	268	WT	WT
173	WT	WT	221	WT	WT	269	WT	WT
174	Mutant	Mutant	222	WT	WT	270	WT	WT
175	WT	WT	223	Mutant	Mutant	271	Mutant	Mutant
176	Mutant	Mutant	224	Mutant	Mutant	272	WT	WT
177	WT	WT	225	WT	WT	273	WT	WT
178	Mutant	Mutant	226	WT	WT	274	WT	WT
179	WT	WT	227	WT	WT	275	WT	WT
180	WT	WT	228	WT	WT	276	WT	WT
181	Mutant	Mutant	229	WT	WT	277	Mutant	Mutant
182	WT	WT	230	WT	WT	278	WT	WT
183	WT	WT	231	WT	WT	279	Mutant	Mutant
184	WT	WT	232	WT	WT	280	WT	WT
185	Mutant	Mutant	233	Mutant	Mutant	281	WT	WT
186	Mutant	Mutant	234	Mutant	Mutant	282	WT	WT
187	WT	WT	235	Mutant	Mutant	283	WT	WT
188	WT	WT	236	WT	WT			
189	WT	Mutant	237	WT	WT			
190	WT	WT	238	Mutant	Mutant			
191	WT	WT	239	WT	WT			
192	Mutant	Mutant	240	WT	WT			
193	Mutant	Mutant	241	Mutant	Mutant			
194	WT	WT	242	WT	WT			
195	WT	WT	243	WT	WT			
196	WT	WT	244	Mutant	Mutant			
197	Mutant	Mutant	245	Mutant	Mutant			
198	WT	WT	246	WT	WT			
199	WT	WT	247	Mutant	Mutant			
200	WT	WT	248	Mutant	Mutant			
201	WT	WT	249	Mutant	Mutant			
202	WT	WT	250	Mutant	Mutant			
203	Mutant	Mutant	251	WT	WT			
204	WT	WT	252	Mutant	Mutant			
205	Mutant	Mutant	253	Mutant	Mutant			
206	WT	WT	254	WT	WT			
207	WT	WT	255	WT	WT			
208	Mutant	Mutant	256	WT	WT			
209	WT	WT	257	WT	WT			
210	WT	WT	258	WT	WT			
211	WT	WT	259	WT	WT			
212	Mutant	Mutant	260	Mutant	Mutant			
213	WT	WT	261	Mutant	Mutant			
						**n mutated cases**	**87**	**93**
